# Differences in mental health inequalities based on university attendance: Intersectional multilevel analyses of individual heterogeneity and discriminatory accuracy

**DOI:** 10.1016/j.ssmph.2022.101149

**Published:** 2022-06-18

**Authors:** Kieran Balloo, Anesa Hosein, Nicola Byrom, Cecilia A. Essau

**Affiliations:** a USQ College, University of Southern Queensland, Springfield, Queensland, Australia; b Surrey Institute of Education, University of Surrey, Guildford, Surrey, UK; c Department of Psychology, Institute of Psychiatry, Psychology and Neuroscience, King's College London, London, UK; d School of Psychology, University of Roehampton, London, UK

**Keywords:** Young People's mental health, Mental distress, Health equity, MAIHDA, Higher education, Intersectionality

## Abstract

There is an increasing focus on structural and social determinants of inequalities in young people's mental health across different social contexts. Taking higher education as a specific social context, it is unclear whether university attendance shapes the impact of intersectional social identities and positions on young people's mental health outcomes. Multilevel Analysis of Individual Heterogeneity and Discriminatory Accuracy (MAIHDA) was used to predict the odds that mental distress during adolescence, sex, socioeconomic status, sexual identity, ethnicity, and their intersections, were associated with young people's mental health outcomes at age 25, and whether this differed based on university attendance. Data from the Longitudinal Study of Young People in England cohort study were analysed with the MAIHDA approach, and the results did not reveal any evidence of multiplicative intersectional (i.e., aggravating) effects on young people's mental health outcomes. However, important main effects of social identities and positions (i.e., an additive model) were observed. The findings suggested that being female or identifying as a sexual minority increased the odds of young people experiencing mental health problems at age 25, although the odds of self-harming were half the size for sexual minorities who had attended university. Black and Asian individuals were less likely to declare a mental illness than White individuals. Young people who grew up in a more deprived area and had not attended university were more likely to experience mental health problems. These findings imply that mental health interventions for young people do not necessarily have to be designed exclusively for specific intersectional groups. Further, university attendance appears to produce better mental health outcomes for some young people, hence more investigation is needed to understand what universities do for young people, and whether this could be replicated in the wider general population.

## Highlights

• MAIHDA was used to analyse young people's mental health outcomes at age 25.
• Intersectional effects were examined both in and out of the university context.
• There was no evidence for multiplicative effects on young people's mental health.
• Additive models were most suitable for understanding mental health inequalities.
• Interventions might be best targeted at broad social group memberships.

## 1 Introduction

Experiencing mental health problems early in life can lead to profound adverse consequences for an individual's mental health outcomes in adulthood (Essau et al., 2014), with the potential for further negative impacts on their educational and employment life outcomes (Frijters et al., 2014; Hale & Viner, 2018). These mental health trajectories are also shaped by social group memberships, including social identities and social positions. For example, there may be a heightened risk of poor mental health and wellbeing outcomes for women (Rosenfield & Smith, 2012; Thorley, 2017), individuals from a low socioeconomic status (SES) background (Cosco et al., 2016; McLaughlin et al., 2012), individuals identifying as LGBT or a sexual minority (Plöderl & Tremblay, 2015; Russell & Fish, 2016), and those from an ethnic minority background (Stevenson & Rao, 2014). From a population health perspective, incidence of disease and poor health is influenced by social inequity or social policies (McAllister et al., 2018) and structural discrimination (Krieger, 2014) that advantage or disadvantage particular groups (Bauer, 2014; Rose, 1985). For example, trauma exposure and victimisation of Black individuals increases their risk of psychosis (Harnett & Ressler, 2021). Thus, social identities and positions might in fact be seen as proxies for systemic marginalisation, such as sexism, racism, homophobia, and classism (Dhamoon & Hankivsky, 2011; Evans, 2019a). In essence, although social identities and positions might occur at the individual level, their impact on predicting health outcomes is shaped by macro level factors:

oppressive social relations (e.g., structural racism) are expressed in political, social, and economic processes that create unequal living and working conditions and harm the health of marginalized groups through multiple “pathways of embodiment,” including social and economic deprivation, toxic/hazardous living conditions, social trauma, and inadequate healthcare (Homan, 2019, p. 492).

In the current study, we examine how social identities and positions, and their intersections, predict the mental health outcomes of young people, with a particular focus on whether there are differences in relationships based on university attendance.

## 2 Intersectionality and Multilevel Analysis of Individual Heterogeneity and Discriminatory Accuracy (MAIHDA)

Influenced by Black feminism, Kimberlé Crenshaw introduced the term *intersectionality* to argue that disadvantage does not occur along a “single-axis framework” (Crenshaw, 1989, p. 140) of individual social identities, such as race or sex in isolation. Instead, Crenshaw (1991) examined how being a member of multiple social categories can help explain discrimination, such as comparing the experiences of Black women with those of White males. An intersectional perspective reinforces the position that health inequalities are a result of the structural power hierarchies that shape individuals’ experiences (Dhamoon & Hankivsky, 2011). Intersectionality has traditionally been explored within a qualitative paradigm (Bauer et al., 2021). However, recently, intersectional scholars have used existing social identities and positions (e.g., sex, ethnicity, etc.) as provisional analytical categories (McCall, 2005) to draw on quantitative analyses (Codiroli Mcmaster & Cook, 2019), which has been found to be particularly beneficial within a population health context (Bauer, 2014). Quantitative approaches make it possible to determine whether multiple memberships of marginalised groups combine to have a cumulative or aggravating negative effect on health outcomes (Kern et al., 2020). A cumulative effect in which the social identities and positions act independently, is known as an additive model, whereas an aggravating effect in which there are interactions between categories indicating that characteristics multiply and amplify each other, is known as a multiplicative model (Kern et al., 2020). Adopting existing categories of social identities and positions for exploring intersectionality was termed by McCall (2005) as *intercategorical complexity*, so this approach lends itself to quantitative analyses. This contrasts with *anticategorical complexity*, which rejects the notion that social life can be reduced to categories, and *intracategorical complexity*, which focuses on inequalities within, rather than between, social groups (McCall, 2005).

While single-level regression analyses have traditionally been used to construct interaction terms for evidence of intersectionality (Bell et al., 2019), Evans et al. (2018) proposed a multilevel approach to analysing quantitative intersectional data, which is considered to be “the new gold standard for investigating health disparities” (Merlo, 2018, p. 79). Known as Multilevel Analysis of Individual Heterogeneity and Discriminatory Accuracy (MAIHDA) (Merlo, 2018), Evans et al. (2018) outlined a number of advantages to this approach over single-level regressions. Firstly, estimates are adjusted to account for the sample size within a particular social stratum (social strata are the points of intersection between social identities). Therefore, MAIHDA has been recommended as the preferred intersectional analytic approach (for binary outcomes) when sample sizes are small and there are a large number of intersections (Mahendran et al., 2022). Secondly, interpretability can be increased through the use of graphs comparing the different outcomes across the social strata. These graphs also enable comparisons across combinations of privilege and marginalisation (e.g., low SES white males vs. high SES Black females), rather than simply one combination of privilege as the reference point for all other combinations (Evans et al., 2018). Finally, multilevel modelling positions the intersection between categories at the level of the social system rather than the social identity or position, which fits more closely with intersectionality theory: “intersectionality considers the interaction of such categories as organizing structures of society, recognizing that these key components influence political access, equality, and the potential for any form of justice” (Hancock, 2007, p. 64).

The current mental health inequalities discourse highlights an interest in how intersectionality can be used to understand mental health outcomes (Fagrell Trygg et al., 2019). Thus far, the few studies using the MAIHDA approach for investigating mental health outcomes within an intersectional framework have found little evidence that inequalities are predominantly explained by a multiplicative model. For example, the first MAIHDA study examining mental health inequalities found that the majority of between-strata variance in depression could be explained by an additive model (Evans & Erickson, 2019). That is, the negative impact of membership of marginalised social groups was cumulative. Fagrell Trygg et al. (2019) note that some intersections may only hold relevance and meaning within certain population group contexts. Therefore, the roles of intersectional identities might benefit from being explored through the lenses of different social contexts (Evans, 2019b; Ghavami et al., 2016). Indeed, when drawing on data collapsed across multiple countries, Kern et al. (2020) found no evidence for multiplicative intersectional effects on adolescent mental wellbeing (life dissatisfaction and psychosomatic complaints), but when they considered variation in national contexts, they found evidence for more negative impacts on mental wellbeing for the multiply marginalised in some countries only. Thus, multiplicative models may still hold further explanatory power for understanding mental health inequalities within certain social contexts that are yet to be explored.

## 3 University as a social context

Worldwide, there are increasing concerns about the mental health of university students. An international survey found that over a third of students reported a lifetime disorder (Auerbach et al., 2018). Students are exposed to a particular set of psychosocial stressors and pressures to participate in risky behaviours (e.g., binge drinking and use of recreational drugs), which increase their risks of developing a mental health problem (Duffy et al., 2019). With around 75% of people experiencing a problem by age 24 (Jones, 2013), the period when the majority of students attend university (i.e., during late adolescence and young adulthood) occurs during a critical developmental stage. In England, over 50% of young people now participate in higher education (Department for Education, 2021), so university is an important social context that could be impacting on young people's mental health outcomes. University may also play a role in shaping mental health inequalities; although universities might aspire to increase opportunities for upward social mobility, they may simultaneously reinforce and strengthen dominant societal modes of elitism, privilege and inequality (Brennan & Naidoo, 2008). Despite this, intersectional frameworks for understanding this context are underexplored. Therefore, the current study uses the university as a social context, comparing outcomes for both those who had attended university and those who had not.

It is also still not clear whether there are longer-term effects of university attendance on mental health outcomes. With the move towards university-based mental health and wellbeing interventions (Byrom, 2018), it is vital to understand whether certain intersectional groups are more in need of targeted approaches within the university space. That is, are the multiply marginalised more likely to have negative or positive mental health outcomes as a result of having been to university? This is important to understand, since targeted intervention risks stigmatisation if there is no evidence for those particular social groups being more in need of targeted support (Bauer & Scheim, 2019; Hernández-Yumar et al., 2018). Mental health stigma leads to negative stereotypes that can affect an individual's quality of life, and intersectional stigma has a compounding effect (Hermaszewska et al., 2022).

## 4 The present study

In the current study, an intercategorical approach to intersectionality was adopted, and MAIHDA analyses (Evans et al., 2018; Merlo, 2018) were performed to predict the odds of young people having mental health problems at age 25 based on the intersection of social identities and positions known to be associated with mental health problems (i.e., sex, SES, sexual identity and ethnicity). By the time this developmental stage occurs, it is anticipated that it would be possible to ascertain the longer-term effects of university on mental health outcomes. Additionally, since subjective social status has been found to be associated with ill-health (Singh-Manoux et al., 2003), we postulated that having a history of mental health problems might mean that it becomes intrinsically tied to a young person's social identity and/or position. Therefore, we also positioned experience of mental distress during adolescence as a social category in these intersectional analyses. Finally, in order to understand the role of university, as a social context, on shaping the impact of social identities and positions, and their intersections, these analyses were performed separately for those who had attended university and those who had not. Hence, we intended to answer the call for more quantitative intersectional research that considers the roles of different environmental social contexts (Evans, 2019b). The aim was to explore whether differences between social strata (i.e., a multiplicative model) explain mental health outcomes better than independent social identities and positions (i.e., an additive model). Thus, the research questions were:

1. Does the university context shape any multiplicative effects of social identities and positions on longer-term mental health outcomes?
2. Does the university context shape any additive effects of social identities and positions on longer-term mental health outcomes?

## 5 Methods

### 5.1 Data and sample

Survey responses from a representative panel study, the *Longitudinal Study of Young People in England* (LSYPE) (University College London, UCL Institute of Education, Centre for Longitudinal Studies, 2020), were analysed. Respondents (*N* = 15,770) were born in England in 1989–90, then followed up annually over seven recruitment sweeps between 2004 and 2010 (Waves 1–7; 14–20 years old), and again in 2015 (Wave 8; 25 years old). In Wave 4 there was also a boost sample of 352 respondents. In order to create combinations of social identities and positions (i.e., social strata) for the MAIHDA analyses, only participants for whom responses were available for all identity/position variables were included. Therefore, out of the 16,122 respondents who were part of the full LSYPE cohort (original data set plus the boost sample in Wave 4), 10,374 were excluded listwise from the model, with the main reason for this being due to the fact that some of the variables (e.g., university attendance and sexual identity) were recorded during a later recruitment sweep by which point there had been substantial attrition in participation in the LSYPE. This resulted in a sample size of 2605 for those who had not attended university and 2791 for those who had attended university. Table 1 displays a breakdown of descriptive statistics of the sample, taking into account listwise deletion of missing data.

**Table 1:** Descriptive statistics for social strata dimensions of the sample.

	No University		University	
Dimension of social identity or position	*N*	%	*N*	%
Total	2605	100	2791	100
Adolescent mental distress (GHQ) at ages 15 and 17				
No mental distress at both ages	1214	46.6	1202	43.1
Mental distress at either age	1391	53.4	1589	56.9
Sex				
Male	1216	46.7	1179	42.2
Female	1389	53.3	1612	57.8
Social deprivation (IDACI)				
Lowest social deprivation	916	35.2	1322	47.4
Medium social deprivation	912	35.0	895	32.1
Highest social deprivation	777	29.8	574	20.6
Sexual identity				
Heterosexual/straight	2456	94.3	2620	93.9
Sexual minority	149	5.7	171	6.1
Ethnicity				
White	2047	78.6	1875	67.2
Black	106	4.1	186	6.7
Asian	324	12.4	564	20.2
Other Ethnic Group (including Mixed)	128	4.9	166	5.9

### 5.2 Social identities/positions and the social context

The 12-item short version of the General Health Questionnaire (GHQ) (Goldberg & Williams, 1988) was used as a measure of adolescent mental distress at two time-points: age 15 and age 17. Each item uses a Likert response scale from 0 to 3 (e.g., *not at all* to *much more than usual*), which were then summed for a score of between 0 and 36 (higher scores indicating greater mental distress). The GHQ can be used as a screening tool for minor diagnosable psychiatric disorders in the general population, with the 11/12 threshold having the optimum sensitivity and specificity when scored using the above Likert response scoring method (Lundin et al., 2016). Therefore, scores of 12 and above were used to indicate a case of probable diagnosable mental health problem. Cronbach's alpha values showed the scale to have good levels of reliability at age 15 (α = 0.87) and age 17 (α = 0.86). Two groups were created based on the GHQ cut-offs: *No mental distress at both ages 15 or 17* (i.e., GHQ score of 0–11 at both time-points); and *mental distress at either ages 15 or 17* (i.e., GHQ score of 12+ at either time-point).

Biological sex was coded as *male* or *female* based on participants’ survey responses at the earliest time-point this variable was available (Waves 1–8).

A binary variable for sexual identity was computed based on whether the respondent identified as *heterosexual/straight* or a *sexual minority*, the latter category consisting of the following responses: Gay/lesbian, bisexual, or other. This variable was based on the latest given response by the respondent from Waves 6, 7, or 8 (i.e., if the response was not available in the most recent sweep, the earlier response was used, but if it changed, the most recent response was used).

Ethnicity included four groups: *White*, *Black*, *Asian*, or *Other Ethnic Group (including Mixed)*. Ethnicity was predominantly taken from responses during Wave 1, but if not present, the response from Waves 2, 4, or 8 were used.

Social deprivation was used as a measure of SES. It was based on the *Income Deprivation Affecting Children Index* (IDACI), which is a geographical indicator of whether the respondent grew up in an area with a larger proportion of children under 16 years old who live in a low-income household. A geographical SES indicator was used to represent the structural inequalities of mental health outcomes, as an individual's mental health and wellbeing may be affected by the extent of their neighbourhood poverty and disadvantage (Graif et al., 2016; Ludwig et al., 2012). In order to use this continuous variable as a part of the social strata, a tertile split based on responses from the overall LSYPE data set was used to create three categories: *lowest deprivation* (individuals who had an IDACI score in the bottom tertile of all scores across respondents in the full LSYPE data set); *medium deprivation* (IDACI score in the middle tertile) and *highest deprivation* (IDACI score in the top tertile). Three or more categories are seen as preferable to only two categories, because this enables the slope representing the relationship between the predictor and outcome variables for the low vs. medium comparison to be different from the slope for the medium vs. high comparison (DeCoster et al., 2011). Tertile splits are also often used in MAIHDA research in order to create categories for intersections (e.g., Axelsson Fisk et al., 2018; Holman et al., 2020; Kern et al., 2020; Khalaf et al., 2020; Persmark et al., 2019; Wemrell et al., 2021). The full data set was used for creating this split, because recruitment of the complete cohort involved stratified sampling across all regions of England, so it should have been representative of the population of young people at the time it was collected. IDACI was measured at Waves 2 and 3. Responses from Wave 2 were used, but if missing and present at Wave 3, the responses from the later time-point were used.

University attendance was a binary variable representing the social context, based on whether the respondent had been to university or not by age 25.

### 5.3 Adulthood mental health problems at age 25

Three mental health outcomes were taken from Wave 8 responses to the LSYPE survey: mental distress, chronic mental illness, and self-harm. Firstly, respondents completed the GHQ at age 25 (α = 0.90) and two groups were created based on the same cut-offs used during adolescence: *No mental distress at age 25* or *mental distress at age 25*. Secondly, respondents declared whether they had a longstanding illness and reported whether this illness was related to mental health. This was taken as a measure of whether they had declared a chronic mental illness or not at age 25. Thirdly, respondents reported whether they had self-harmed on purpose in the past year at age 25.

### 5.4 Social strata

A Stratum ID variable was constructed for the strata to indicate the intersectional group membership for each respondent, which is necessary for fitting the multilevel models (as discussed below). As outlined above, there were two categories for adolescent mental distress, two categories for sex, three categories for social deprivation, two categories for sexual identity and four categories for ethnicity. Therefore, for example, the ID code 22123 represents respondents who experienced mental distress at either ages 15 or 17 (2), are female (2), from an area with the lowest social deprivation (1), identify as a sexual minority (2), and of Asian ethnicity (3). By combining all combinations of social identity and position categories, there were 96 possible intersectional social strata (i.e., 2 × 2 × 3 × 2 × 4 = 96). However, due to there being no respondents in the LSYPE matching certain intersectional group memberships, there were 69 strata with responses to the outcome variables (i.e., adulthood mental health problems at age 25) for those who had not attended university, and 79 strata for those who had attended university.

### 5.5 Statistical analyses

MAIHDA analyses involve the fitting of multilevel models whereby social strata (as denoted by the stratum ID variable) are placed at level 2 and individual respondents are nested within this at level 1 (Evans et al., 2018). The total variance in the outcome is partitioned into between-strata (i.e., between intersections of identities/positions) and within-strata variance (i.e., within intersections of identities/positions). A null model (with no main effects included) is first produced to calculate a Variance Partition Coefficient (VPC). The VPC can be used in a similar manner to an *R*^2^ model fit statistic to determine the extent to which the social strata can predict scores on the outcome variable (Kern et al., 2020). The VPC of the null model is a measure of the discriminatory accuracy of the different intersectional strata (Axelsson Fisk et al., 2018; Evans et al., 2018). Mathematically, the VPC is analogous to an intra-class correlation coefficient (ICC), which expresses the correlation in scores on the outcome variable between individuals within a cluster. Interpreted as a VPC, a greater ICC value indicates more between-strata variability in the outcome variable, with less variability being explained by differences between individuals nested within strata (Evans, 2019a; Holman & Walker, 2021; Merlo, 2018). Axelsson Fisk et al. (2018) proposed the following grading scale for assessing VPC values, which are multiplied by 100 and expressed as percentages (0–100): non-existent (0–1), poor (>1 to ≤ 5), fair (>5 to ≤ 10), good (>10 to ≤ 20), very good (>20 to ≤ 30), and excellent (>30) differentiation between strata. The VPC is equal to the between-strata variance ($\sigma_{u}^{2}$) divided by the total variance. Total variance is the sum of the between- and within-strata variance ($\sigma_{u}^{2}+\sigma_{e}^{2}$). In the current study, logistic regression models were used because all outcomes were binary. Hence, the within-strata variance ($\sigma_{e}^{2}$) value is equal to the variance of the standard logistic distribution which is π^2^/3 (Goldstein et al., 2002), and can be substituted in the following equation:

$$VPC\equiv ICC=\frac{\sigma_{u}^{2}}{\sigma_{u}^{2}+\sigma_{e}^{2}}=\frac{\sigma_{u}^{2}}{\sigma_{u}^{2}+\frac{\pi^{2}}{3}}$$

Since the VPC is calculated for the null model, the main effects and interaction effects are conflated, which means it is not clear how much of the variability in the outcome is explained by the additive models (i.e., the main effects) and how much by the multiplicative models (i.e., the interactions). Therefore, a model that includes main effects only is then produced to determine whether the additive main effects of the social strata (i.e., fixed effects) can explain variance in the outcome variable. The Proportional Change in Variance (PCV) value is used to estimate what percentage of variance in the VPC is accounted for by the additive main effects. A PCV value is calculated based on the difference in between-strata variance of the null and main effects models. The PCV is calculated by deducting the variance between strata for the main effects model from the between-strata variance for the null model, then dividing this by the between-strata variance for the null model:

$$PCV=\frac{\sigma_{u\left(null\;model\right)}^{2}\;-\;\sigma_{u\left(main\;effects\;model\right)}^{2}}{\sigma_{u\left(null\;model\right)}^{2}}$$

PCV values are also multiplied by 100 and presented as percentages, with a high score indicating that the between-strata variability is mostly explained by the main effects, and a low score suggesting it may be mostly explained by interactions between social strata. Finally, where the PCV values indicate that the between-strata variability is being mostly explained by interaction effects, an examination of the strata-level residuals shows the extent to which the predicted score for each stratum differs from the expected score based on the additive main effects. In order to do this, the expected incidences of the outcome based on the additive main effects are subtracted from the aforementioned predicted scores, which results in a difference known as the strata-level residual. Negative residuals indicate that incidences for the stratum are lower than would be expected based on the additive main effects, whereas positive residuals are higher than expected. That is, the residual shows how much an interaction effect (i.e., the combination of multiple identities/positions) differs from what is explained by the main effects alone (i.e., can interactions explain the outcome better than the main effects?). If the 95% credible intervals for the residual do not cross zero, these effects are considered to be statistically significant. The MAIHDA approach down-weights the residuals for intersections with small samples, so these social strata will not have a disproportionate effect on the results (Bell et al., 2019; Mahendran et al., 2022).

All multilevel models were fit using MLwiN 3.02 (Rasbash et al., 2020) called from Stata 16.1 using the *runmlwin* command (Leckie & Charlton, 2012). Following the same estimation approaches and options used in many previous MAIHDA analyses (e.g., Evans et al., 2018), all analyses used Bayesian Markov Chain Monte Carlo (MCMC) estimation (Browne, 2019) with diffuse (non-informative) priors. The burn-in phase was 5000 iterations with a total length of 50,000 iterations, and thinning every 50 iterations. Stata syntax was adapted from Axelsson Fisk et al. (2018) to fit the models and obtain 95% credible intervals around estimates.

## 6 Results

### 6.1 Research question 1: Does the university context shape any multiplicative effects of social identities and positions on longer-term mental health outcomes?

**Fig. 1:**
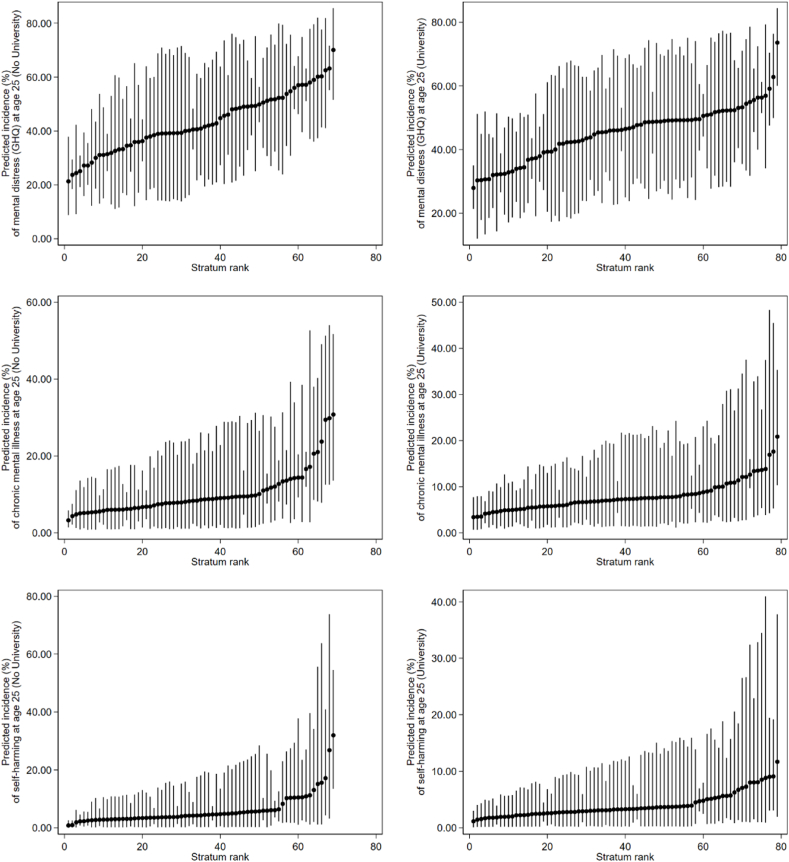
Predicted incidence (%) of mental health problems by social strata for the null models (main effects and interaction effects conflated). The black circles represent the predicted incidence in that particular social stratum. The vertical lines are 95% credible intervals. Strata have been ranked from intersections with the lowest to highest incidence rates.

**Fig. 2:**
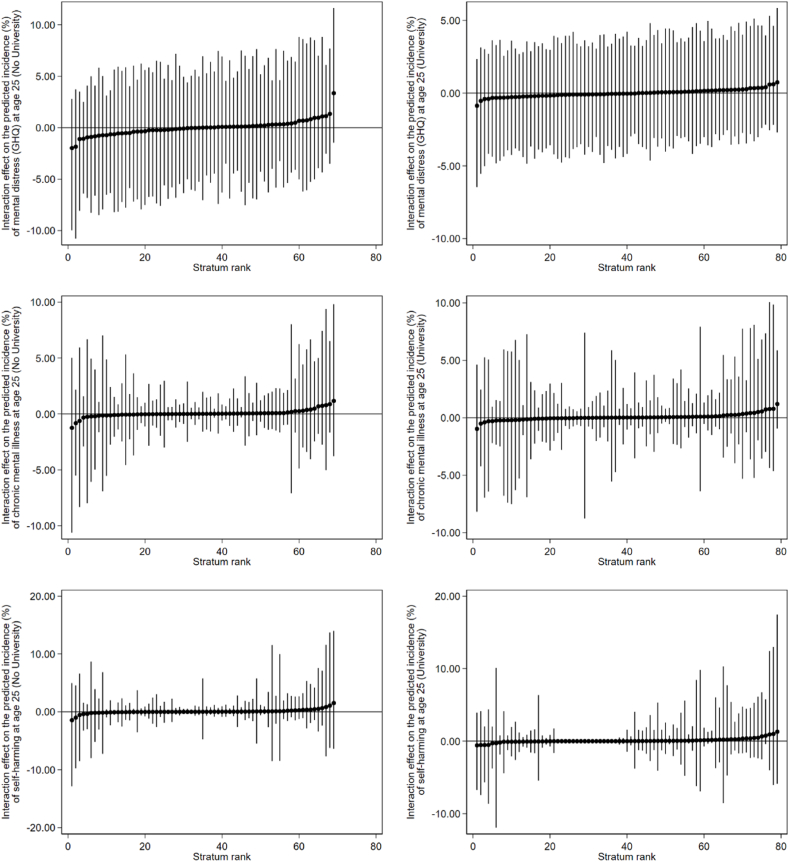
Intersectional effects on the predicted incidence (%) of mental health problems by social strata. The black circles represent the predicted incidence in that particular social stratum based on the interaction effects minus the main effects, which is represented by the horizontal line. The vertical lines are 95% credible intervals. Strata have been ranked according to the extent to which each interaction effect differs from what is explained by the main effects alone.

Before taking into account the main effects, the VPC values for the null models (see Table 2) suggested fair to excellent levels of between-stratum differences occurring at the intersectional strata level. This is supported by the graphs in Fig. 1, which display the predicted incidences of mental health problems at age 25 by social strata. In the graphs in Fig. 1, the predicted incidences (as represented by the black circles) are different for each stratum, indicating that there is variability occurring between strata. After taking into account the main effects, the PCV values were all above 90% (see Table 2), indicating that the main effects accounted for the majority of this variance for each outcome. Therefore, the additive models appeared to explain most of the differences in incidence of adulthood mental health problems between strata (as are extrapolated further below under Research Question 2). Fig. 2 displays the strata-level residuals (i.e., the extent to which each social stratum differed from what was explained by the main effects alone for each of the outcomes). In-line with what was suggested by the large PCV values, the 95% credible intervals for each of the intersectional effects cross zero, so they are all non-significant. Therefore, no evidence of multiplicative intersectional effects could be ascertained from these analyses. This means that incidences of adulthood mental health problems (i.e., mental distress, chronic mental illness, and self-harm) are better explained by the main effects than the interaction effects for both those who had attended university and those who had not. Tables A1–A6 in Appendix A include the predicted incidence scores within each social stratum for each model, and can be used to identify the different strata in Fig. 1, Fig. 2.

**Table 2:** MAIHDA models predicting likelihood of experiencing mental distress, declaring chronic mental illness, or declaring self-harm in the last year (at age 25), split by university attendance.

	Adulthood mental health problems at age 25					
	**Mental distress (GHQ)**		**Chronic mental illness**		**Self-harmed**	
	**No University** ***N* = 2497**	**University** ***N* = 2736**	**No University** ***N* = 2534**	**University** ***N* = 2751**	**No University** ***N* = 2470**	**University** ***N* = 2719**
**Main Effects Model**	**OR (95% CI)**	**OR (95% CI)**	**OR (95% CI)**	**OR (95% CI)**	**OR (95% CI)**	**OR (95% CI)**
Intercept	0.28 (0.22, 0.36)*	0.45 (0.37, 0.54)*	0.04 (0.02, 0.06)*	0.05 (0.04, 0.08)*	0.01 (0.01, 0.02)*	0.01 (0.01, 0.03)*
Adolescent mental distress at either ages 15 or 17 (GHQ) (Ref: No mental distress at both ages 15 or 17)	3.06 (2.50, 3.79)*	2.31 (1.93, 2.76)*	2.61 (1.82, 3.61)*	1.40 (0.96, 1.95)	3.46 (1.99, 5.78)*	2.87 (1.63, 5.24)*
Female (Ref: Male)	1.31 (1.06, 1.59)*	1.31 (1.08, 1.54)*	1.43 (1.01, 1.96)*	1.52 (1.04, 2.14)*	1.45 (0.89, 2.24)	1.33 (0.76, 2.09)
Social deprivation (IDACI) (Ref: Lowest deprivation)						
Medium deprivation	1.37 (1.04, 1.73)*	0.84 (0.68, 1.03)	1.47 (0.95, 2.03)	0.88 (0.58, 1.32)	1.32 (0.73, 2.17)	0.85 (0.44, 1.48)
Highest deprivation	1.39 (1.05, 1.79)*	0.94 (0.72, 1.18)	1.55 (1.00, 2.26)*	1.22 (0.74, 1.99)	1.18 (0.63, 2.06)	1.08 (0.50, 2.01)
Sexual minority (Ref: Heterosexual/straight)	2.14 (1.44, 3.03)*	2.15 (1.50, 2.99)*	3.87 (2.50, 5.88)*	4.02 (2.52, 6.07)*	7.19 (3.86, 11.74)*	3.85 (2.01, 6.78)*
Ethnicity (Ref: White)						
Black	1.06 (0.65, 1.60)	0.92 (0.63, 1.29)	0.29 (0.07, 0.67)*	0.32 (0.11, 0.73)*	0.96 (0.24, 2.30)	0.0001 (0.00, 0.00)
Asian	0.75 (0.55, 1.00)	1.00 (0.79, 1.25)	0.33 (0.15, 0.60)*	0.33 (0.17, 0.57)*	0.57 (0.18, 1.23)	0.69 (0.30, 1.28)
Other Ethnic Group (including mixed)	0.77 (0.50, 1.18)	0.94 (0.65, 1.30)	0.64 (0.25, 1.19)	0.76 (0.37, 1.31)	1.02 (0.30, 2.25)	1.84 (0.82, 3.43)
	**Mean (95% CI)**	**Mean (95% CI)**	**Mean (95% CI)**	**Mean (95% CI)**	**Mean (95% CI)**	**Mean (95% CI)**
Between-Strata Variance	0.02 (0.001, 0.07)	0.01 (0.0004, 0.03)	0.03 (0.001, 0.14)	0.03 (0.001, 0.20)	0.05 (0.001, 0.30)	0.07 (0.001, 0.52)
DIC	265.19	310.31	181.20	205.73	153.72	154.36
VPC (%)	0.6	0.2	0.8	1.0	1.5	2.1
**Null Model**	**OR (95% CI)**	**OR (95% CI)**	**OR (95% CI)**	**OR (95% CI)**	**OR (95% CI)**	**OR (95% CI)**
Intercept	0.74 (0.59, 0.93)*	0.82 (0.67, 0.99)*	0.09 (0.06, 0.12)*	0.07 (0.05, 0.09)*	0.04 (0.02, 0.06)*	0.03 (0.02, 0.04)*
	**Mean (95% CI)**	**Mean (95% CI)**	**Mean (95% CI)**	**Mean (95% CI)**	**Mean (95% CI)**	**Mean (95% CI)**
Between-Strata Variance	0.48 (0.24, 0.86)	0.31 (0.15, 0.61)	0.81 (0.36, 1.58)	0.56 (0.15, 1.29)	1.49 (0.53, 3.06)	1.02 (0.14, 2.86)
DIC	294.25	338.89	206.53	239.63	171.94	177.88
VPC (%)	12.7	8.6	19.7	14.5	31.2	23.7
PCV (%)	96.1	97.8	96.8	94.2	96.6	93.0

*Note.* *95% credible intervals do not cross one, so effect is significant. OR = Odds Ratio. The Deviance Information Criterion (DIC) is used as a goodness-of-fit measure for Bayesian multilevel models; lower DIC scores indicate a better fit. Both the VPC and PCV values have been multiplied by 100 and presented as percentages.

### 6.2 Research question 2: Does the university context shape any additive effects of social identities and positions on longer-term mental health outcomes?

Focusing on the additive main effects only (Table 2), there were some differences in main effects based on university context. For those who had not attended university, experiencing mental distress during adolescence led to a greater likelihood of experiencing mental distress (at age 25), declaring a chronic mental illness, or reporting self-harm in the last year (3.1, 2.6, and 3.5 times greater than those who did not experience mental distress during adolescence, respectively). For those who had attended university, experiencing mental distress during adolescence still led to significantly greater odds of experiencing mental distress (at age 25) or reporting self-harm in the last year, although the sizes of these odds were smaller (2.3 vs. 3.1 times and 2.9 vs. 3.5 times, respectively). However, the odds of declaring a chronic mental illness among those who experienced mental distress during adolescence and had attended university were non-significant.

Females were significantly more likely to experience mental distress (at age 25) and declare a chronic mental illness than males, for both those who had attended university (1.3 and 1.5 times, respectively) and those who had not (1.3 and 1.4 times, respectively).

For those who had not attended university, respondents who had the medium or highest deprivation levels (IDACI) had a greater likelihood of experiencing mental distress (1.4 times for both compared to the lowest IDACI tertile). These same respondents who had the highest deprivation levels also had significantly greater odds of declaring a chronic mental illness (1.6 times). However, for those who had attended university, there were no significant associations between social deprivation and mental health problems.

Sexual minority respondents were significantly more likely to experience mental health problems than heterosexual/straight respondents regardless of university attendance (2.1–7.2 times). Although sexual minority respondents who had not attended university had far greater odds of reporting having self-harmed than those who had attended university (7.2 vs. 3.9 times).

Finally, Black and Asian respondents were less likely to declare a chronic mental illness than White respondents, regardless of university attendance (3.0–3.4 times).

## 7 Discussion

The current study aimed to ascertain whether the social context of university has an effect on shaping mental health inequalities, and whether such inequalities are multiplicative, additive, or both. For young people who held particular group memberships, the findings suggested that they were more likely to have better mental health outcomes if they had attended university. Analyses did not reveal any evidence of multiplicative intersectional effects, which is consistent with many other MAIHDA studies exploring various health inequalities (Holman et al., 2020). Therefore, social identities and positions do not appear to amplify each other in their predictions of mental health problems at age 25. Instead, these social dimensions are layered and independent, so based on the current analyses, additive models appear to be most suitable for understanding mental health inequalities in young people. Similar, to Evans and Erickson's (2019) findings on depression among adolescents and young adults, the current study's findings suggest that interactions of social identities may not be appropriate for predicting longer-term mental health outcomes within certain contexts. Thus, the main social identities of young people that we investigated may help explain mental health inequalities better, but we need to be cautious when interpreting the impact of multiple identities. Hence, we will now focus on the additive model results.

There were some differences in main effects based on university attendance. For respondents who had not attended university, experiencing mental distress during adolescence, being female, growing up in a more deprived area, and identifying as a sexual minority all appeared to increase the odds of experiencing mental distress at age 25. Not all of these main effects were present for those who had attended university. Females were more likely to experience mental distress or declare a mental illness than males, regardless of the university context. This is consistent with previous findings showing that females are more likely to experience internalising problems (anxiety and depression) (Rosenfield & Smith, 2012).

There was a lack of association between experiences of adolescent mental distress and declarations of chronic mental illness at age 25 for those who had attended university (despite a relationship being present for those who had not attended university). This suggests that the university environment could be having a positive effect on outcomes even for those with a history of mental distress. On the one hand, it may be the case that young people who have not experienced mental distress during adolescence are more likely to attend university. On the other hand, 56.9% of respondents who experienced mental distress during their adolescence also attended university, compared to 53.4% of those who had not attended (see Table 1). Hence, this may suggest that universities are “reducing” the mental distress faced by young people. The mechanisms for how this is occurring are unclear, but it may be related to the sense of community, social networks, realisation of life goals or supportive culture of university. Policies have existed for some time that emphasise the benefits of universities fostering social cohesion and a sense of belonging among students, with the potential for positive impacts on their wellbeing (Ahn & Davis, 2020; Hughes & Spanner, 2019; Mountford-Zimdars et al., 2015). Furthermore, increasing efforts to embed mental health and wellbeing support at university (e.g., Byrom, 2018) could be having a longer-term benefit on graduates’ mental health outcomes (e.g., through increasing resilience). University environments could therefore act as a protective factor against mental health problems.

For those who had attended university, growing up in a more deprived area did not predict mental distress at age 25. It is unclear why this was the case, but one argument is that those from areas with the highest levels of deprivation were less likely to attend university and that is why there was no association. Indeed, within the university population, our descriptive statistics showed that 20.6% were from the highest deprivation group, whereas outside of university, 29.8% were from this same group. Alternatively, these positive effects could be due to the upward social mobility opportunities afforded by higher education rather than the environment specifically, since university education might reduce some of the economic disparities that lead to unequal health outcomes. For example, austerity measures that disproportionately impact on the most deprived groups are associated with poorer mental health outcomes (McAllister et al., 2018). However, individuals from a low SES background are also more likely to experience traumatic events that lead to mental distress, and these might occur early in life (Ashton et al., 2016; Hatch & Dohrenwend, 2007; Sweeney & Taggart, 2018). Therefore, the reduction in disadvantage that potentially results from a university education might not be enough to counter pre-existing mental distress from childhood and adolescence.

Interestingly, despite previous research showing that ethnic minority individuals are at a greater risk of experiencing mental health problems (Harnett & Ressler, 2021), the current findings revealed that both Black and Asian individuals were less likely to declare a mental illness than White individuals, regardless of whether they had attended university. However, African-Caribbean groups in the UK have been found to experience stigma and negative pathways to accessing mental health services (e.g., police-enforced mandatory attendance at psychiatric services), which can delay their help-seeking compared to White individuals (Mantovani et al., 2017; Morgan et al., 2004). Similarly, South Asian individuals have conveyed reluctance sharing concerns at UK-based mental health services due to the perception that there will be a lack of sensitivity to their cultural needs (Bowl, 2007). Thus, the lower incidence of declarations of mental illness among Black and Asian respondents in the current study could be due to them not having sought help in the past that might have led to a diagnosis. Indeed, the absence of significant effects for ethnicity on the mental distress (GHQ) measure suggests that Black and Asian individuals may only be faring better than White respondents in terms of having a lower incidence of declaring a mental illness, not necessarily in terms of having fewer mental health problems.

Sexual minority individuals were more likely to experience all types of mental health problems in either the university or non-university context. However, the odds ratios for self-harm were half the size for those who had attended university. For some, higher education is seen as an open and inclusive environment in which individuals are more free to explore their sexual identities (Formby, 2013, 2015). Scourfield et al. (2008) found that one strategy of resilience for LGBT young people facing homophobia was to move to a gay-friendly safe place like university. In a survey of LGBTQ students in England, many respondents noted that they had seen posters related to LGBTQ issues around campus, which made them feel visible, and 76.5% of them also reported feeling comfortable challenging homophobic, biphobic or transphobic discrimination in the university environment (Grimwood, 2017). Thus, having a space to express their true sexual identities may have longer-term mitigating effects on the risk of self-harming behaviour for some sexual minority individuals.

## 8 Limitations and implications

One of the limitations of the current study is that the sample sizes were relatively small for examining intersectional effects, and some social strata were not present in the sample at all. It is anticipated that the MAIHDA approach will have addressed this issue by down-weighting the residuals for intersections with small samples (Bell et al., 2019; Mahendran et al., 2022). However, it is also possible that the analyses will only have performed as well as (although not poorer than) main effects regressions (Mahendran et al., 2022). Nonetheless, main effects regressions would not have been a useful means of exploring multiplicative intersectional effects, so MAIHDA was the optimal approach for ascertaining the absence of a multiplicative model. The follow-up additive model would then have performed at least as well as a single-level regression analysis. Future studies might benefit from exploring different social identities and positions across new social contexts, drawing on data from larger cohort studies using the MAIHDA approach. It would also be useful to explore the role of different university types to help disentangle whether mental health outcomes differ based on environmental differences or as a result of receiving a university education.

The implications of the current study are that interventions for marginalised groups might be beneficial if they are targeted at the broad social group memberships found to be associated with mental health problems, instead of being targeted at specific intersectional groups. That is, interventions could be designed for, for example, females and sexual minority individuals, rather than specifically targeted at sexual minority females only. This could benefit individuals who hold one or both group memberships and avoids the risk of stigmatisation (Bauer & Scheim, 2019; Hernández-Yumar et al., 2018). This may be a judicious approach until actual evidence has been found for combinations of identities and positions having an aggravating effect on mental health problems within the contexts investigated in this study. However, there is a recognition that sometimes when people share the same social identity or position, they may be able to feel more connection and work together (Haslam et al., 2022). Hence, if interventions are held in-person and in groups, then having interventions based on intersectional identities and positions may be appropriate. Furthermore, since outcomes appeared to be better for some marginalised groups who attended university, it would be useful to understand more about what appears to be benefiting those particular groups of individuals who have been to university. For example, the increase in university-based initiatives for promoting positive mental health and wellbeing may be key to supporting longer-term outcomes, so there is now a question about how these could be replicated within the general population.

## 9 Conclusion

In conclusion, use of the quantitative MAIHDA approach revealed that the university context does not appear to shape any multiplicative intersectional effects of social identities and positions on longer-term mental health outcomes. However, differences in mental health inequalities based on university attendance were found for the additive effects. Better mental health outcomes were found for sexual minorities, and those who grew up in a more deprived area, if they had attended university.

## Declarations

The authors have no relevant financial or non-financial interests to disclose.

## Availability of data

The data that support the findings of this study are available via the UK Data Service at https://doi.org/10.5255/UKDA-SN-5545-7.

## Funding

This work was supported by the 10.13039/501100000269Economic and Social Research Council [grant number ES/T002255/1].

## Credit author statement

Kieran Balloo: Conceptualization; Data curation; Formal analysis; Funding acquisition; Investigation; Methodology; Writing - original draft; Writing - review & editing. Anesa Hosein: Conceptualization; Funding acquisition; Investigation; Methodology; Writing - original draft; Writing - review & editing. Nicola Byrom: Conceptualization; Funding acquisition; Writing - original draft; Writing - review & editing. Cecilia Essau: Conceptualization; Funding acquisition; Writing - original draft; Writing - review & editing.

## Ethical statement

De-identified secondary data only were used to support the findings of this study. A Self-Assessment for Governance and Ethics was undertaken (ID: 514292-514283-59380342), and this confirmed that a full ethics review by the University of Surrey Ethics Committee was not necessary.
