# Supplementary material for: Differences in mental health inequalities based on university attendance: Intersectional multilevel analyses of individual heterogeneity and discriminatory accuracy
Source: SSM Popul Health. 2022 Jun 18;19:101149. doi: 10.1016/j.ssmph.2022.101149 (PMC9253404; doi:10.1016/j.ssmph.2022.101149)
Supplement: Multimedia component 1 [file mmc1.docx]

# Appendix A. Supplementary Data

## Table A1. Predicted incidence (%) of mental distress at age 25 for individuals who did not attend university by social strata based on conflated interaction effects and main effects, main effects only, and differences between intersections and main effects, ranked according to the extent to which each interaction effect differs from what is explained by the main effects alone (i.e. the residuals)

| Stratum | Stratum Label | Number of cases | Number of individuals | Predicted incidence (I+M effects) | Low 95% CI predicted incidence (I+M effects) | High 95% CI predicted incidence (I+M effects) | Predicted incidence (M effects) | Low 95% CI predicted incidence (M effects) | High 95% CI predicted incidence (M effects) | Predicted incidence (I effects) | Low 95% CI predicted incidence (I effects) | High 95% CI predicted incidence (I effects) |
| --- | --- | --- | --- | --- | --- | --- | --- | --- | --- | --- | --- | --- |
| 21111 | Adolescent mental distress at either ages 15 or 17 - Male - Lowest IDACI - Heterosexual/straight - White | 60 | 149 | 43.83 | 37.15 | 49.78 | 45.80 | 40.23 | 53.08 | -1.97 | -9.97 | 2.79 |
| 12221 | No adolescent mental distress at either ages 15 or 17 - Female - Middle IDACI - Sexual minority - White | 2 | 12 | 48.96 | 35.50 | 60.57 | 50.80 | 40.17 | 61.15 | -1.84 | -10.79 | 3.73 |
| 22311 | Adolescent mental distress at either ages 15 or 17 - Female - Highest IDACI - Heterosexual/straight - White | 77 | 135 | 59.06 | 52.86 | 64.87 | 60.15 | 54.07 | 66.50 | -1.09 | -8.08 | 3.51 |
| 11211 | No adolescent mental distress at either ages 15 or 17 - Male - Middle IDACI - Heterosexual/straight - White | 47 | 196 | 26.39 | 21.53 | 31.39 | 27.46 | 22.59 | 31.96 | -1.07 | -6.41 | 2.51 |
| 22111 | Adolescent mental distress at either ages 15 or 17 - Female - Lowest IDACI - Heterosexual/straight - White | 118 | 235 | 51.41 | 46.31 | 56.57 | 52.35 | 47.06 | 57.60 | -0.94 | -6.84 | 4.09 |
| 21311 | Adolescent mental distress at either ages 15 or 17 - Male - Highest IDACI - Heterosexual/straight - White | 37 | 74 | 52.83 | 45.37 | 60.93 | 53.72 | 47.27 | 61.32 | -0.89 | -8.27 | 5.00 |
| 22211 | Adolescent mental distress at either ages 15 or 17 - Female - Middle IDACI - Heterosexual/straight - White | 137 | 237 | 59.00 | 53.48 | 63.89 | 59.83 | 54.45 | 64.86 | -0.83 | -6.60 | 4.09 |
| 22214 | Adolescent mental distress at either ages 15 or 17 - Female - Middle IDACI - Heterosexual/straight - Other (including mixed) | 5 | 13 | 52.12 | 38.88 | 64.11 | 52.88 | 42.27 | 63.78 | -0.76 | -8.50 | 5.82 |
| 22312 | Adolescent mental distress at either ages 15 or 17 - Female - Highest IDACI - Heterosexual/straight - Black | 14 | 26 | 60.15 | 48.58 | 70.37 | 60.87 | 50.84 | 70.87 | -0.72 | -7.95 | 5.03 |
| 11213 | No adolescent mental distress at either ages 15 or 17 - Male - Middle IDACI - Heterosexual/straight - Asian | 1 | 16 | 21.39 | 13.42 | 28.46 | 22.11 | 15.79 | 28.25 | -0.72 | -6.51 | 3.13 |
| 11311 | No adolescent mental distress at either ages 15 or 17 - Male - Highest IDACI - Heterosexual/straight - White | 29 | 113 | 27.11 | 21.75 | 32.81 | 27.74 | 22.96 | 33.50 | -0.64 | -6.35 | 3.63 |
| 22112 | Adolescent mental distress at either ages 15 or 17 - Female - Lowest IDACI - Heterosexual/straight - Black | 1 | 4 | 52.51 | 38.85 | 65.75 | 53.14 | 41.12 | 64.81 | -0.63 | -8.21 | 5.66 |
| 12323 | No adolescent mental distress at either ages 15 or 17 - Female - Highest IDACI - Sexual minority - Asian | 0 | 3 | 43.36 | 29.74 | 55.84 | 43.92 | 32.54 | 55.86 | -0.55 | -8.16 | 5.93 |
| 12212 | No adolescent mental distress at either ages 15 or 17 - Female - Middle IDACI - Heterosexual/straight - Black | 0 | 3 | 33.37 | 21.01 | 45.81 | 33.91 | 23.24 | 44.77 | -0.54 | -7.23 | 5.52 |
| 21113 | Adolescent mental distress at either ages 15 or 17 - Male - Lowest IDACI - Heterosexual/straight - Asian | 2 | 10 | 38.21 | 27.86 | 49.56 | 38.72 | 30.47 | 47.86 | -0.51 | -7.77 | 5.87 |
| 11314 | No adolescent mental distress at either ages 15 or 17 - Male - Highest IDACI - Heterosexual/straight - Other (including mixed) | 1 | 12 | 22.13 | 14.25 | 31.03 | 22.64 | 15.33 | 31.51 | -0.51 | -5.87 | 4.03 |
| 22114 | Adolescent mental distress at either ages 15 or 17 - Female - Lowest IDACI - Heterosexual/straight - Other (including mixed) | 6 | 15 | 44.97 | 33.57 | 57.27 | 45.36 | 35.04 | 56.67 | -0.39 | -7.18 | 6.04 |
| 22324 | Adolescent mental distress at either ages 15 or 17 - Female - Highest IDACI - Sexual minority - Other (including mixed) | 0 | 1 | 69.85 | 56.48 | 81.64 | 70.22 | 58.07 | 81.07 | -0.36 | -6.97 | 4.66 |
| 12222 | No adolescent mental distress at either ages 15 or 17 - Female - Middle IDACI - Sexual minority - Black | 0 | 1 | 51.26 | 35.32 | 67.23 | 51.61 | 37.23 | 66.45 | -0.36 | -7.95 | 6.23 |
| 22212 | Adolescent mental distress at either ages 15 or 17 - Female - Middle IDACI - Heterosexual/straight - Black | 2 | 5 | 60.19 | 46.11 | 71.57 | 60.52 | 48.75 | 70.63 | -0.33 | -7.51 | 5.60 |
| 11222 | No adolescent mental distress at either ages 15 or 17 - Male - Middle IDACI - Sexual minority - Black | 0 | 1 | 44.96 | 29.69 | 61.36 | 45.20 | 31.14 | 59.99 | -0.24 | -6.73 | 5.85 |
| 21121 | Adolescent mental distress at either ages 15 or 17 - Male - Lowest IDACI - Sexual minority - White | 8 | 13 | 63.62 | 53.26 | 74.28 | 63.85 | 54.17 | 72.67 | -0.23 | -6.69 | 5.40 |
| 11323 | No adolescent mental distress at either ages 15 or 17 - Male - Highest IDACI - Sexual minority - Asian | 0 | 1 | 37.42 | 24.54 | 50.50 | 37.65 | 27.17 | 49.58 | -0.23 | -7.37 | 6.50 |
| 21123 | Adolescent mental distress at either ages 15 or 17 - Male - Lowest IDACI - Sexual minority - Asian | 0 | 1 | 56.64 | 42.63 | 69.37 | 56.87 | 44.48 | 68.37 | -0.23 | -7.61 | 6.40 |
| 12213 | No adolescent mental distress at either ages 15 or 17 - Female - Middle IDACI - Heterosexual/straight - Asian | 4 | 17 | 26.74 | 18.34 | 35.38 | 26.95 | 19.64 | 34.44 | -0.21 | -5.60 | 4.77 |
| 11321 | No adolescent mental distress at either ages 15 or 17 - Male - Highest IDACI - Sexual minority - White | 1 | 3 | 44.46 | 32.46 | 56.99 | 44.65 | 33.86 | 55.84 | -0.19 | -6.91 | 6.09 |
| 22321 | Adolescent mental distress at either ages 15 or 17 - Female - Highest IDACI - Sexual minority - White | 8 | 11 | 75.69 | 65.73 | 83.65 | 75.86 | 67.59 | 82.85 | -0.17 | -5.80 | 4.63 |
| 11223 | No adolescent mental distress at either ages 15 or 17 - Male - Middle IDACI - Sexual minority - Asian | 0 | 1 | 37.23 | 24.46 | 50.23 | 37.35 | 26.62 | 49.28 | -0.12 | -6.82 | 7.18 |
| 12314 | No adolescent mental distress at either ages 15 or 17 - Female - Highest IDACI - Heterosexual/straight - Other (including mixed) | 3 | 11 | 27.42 | 18.44 | 38.15 | 27.51 | 19.36 | 36.91 | -0.09 | -5.47 | 6.04 |
| 22322 | Adolescent mental distress at either ages 15 or 17 - Female - Highest IDACI - Sexual minority - Black | 2 | 2 | 76.21 | 63.70 | 86.26 | 76.28 | 65.34 | 85.65 | -0.08 | -6.36 | 4.94 |
| 12311 | No adolescent mental distress at either ages 15 or 17 - Female - Highest IDACI - Heterosexual/straight - White | 27 | 80 | 33.25 | 26.97 | 39.89 | 33.29 | 27.91 | 38.89 | -0.04 | -5.01 | 4.41 |
| 22222 | Adolescent mental distress at either ages 15 or 17 - Female - Middle IDACI - Sexual minority - Black | 1 | 1 | 75.98 | 62.92 | 86.06 | 76.00 | 64.42 | 85.85 | -0.02 | -6.03 | 5.05 |
| 22121 | Adolescent mental distress at either ages 15 or 17 - Female - Lowest IDACI - Sexual minority - White | 12 | 17 | 69.62 | 60.03 | 78.40 | 69.64 | 60.93 | 77.35 | -0.02 | -6.14 | 5.64 |
| 12114 | No adolescent mental distress at either ages 15 or 17 - Female - Lowest IDACI - Heterosexual/straight - Other (including mixed) | 0 | 1 | 21.69 | 13.64 | 31.86 | 21.70 | 14.69 | 30.28 | -0.01 | -5.24 | 4.98 |
| 21212 | Adolescent mental distress at either ages 15 or 17 - Male - Middle IDACI - Heterosexual/straight - Black | 3 | 5 | 54.20 | 40.70 | 66.42 | 54.18 | 42.25 | 65.18 | 0.01 | -7.05 | 6.95 |
| 11112 | No adolescent mental distress at either ages 15 or 17 - Male - Lowest IDACI - Heterosexual/straight - Black | 0 | 2 | 22.67 | 13.77 | 34.28 | 22.65 | 15.05 | 32.04 | 0.02 | -4.64 | 5.29 |
| 11312 | No adolescent mental distress at either ages 15 or 17 - Male - Highest IDACI - Heterosexual/straight - Black | 4 | 15 | 28.60 | 19.59 | 38.94 | 28.57 | 20.40 | 38.17 | 0.03 | -5.35 | 6.40 |
| 12313 | No adolescent mental distress at either ages 15 or 17 - Female - Highest IDACI - Heterosexual/straight - Asian | 11 | 41 | 27.21 | 20.09 | 34.56 | 27.17 | 20.87 | 34.06 | 0.03 | -4.47 | 5.47 |
| 12321 | No adolescent mental distress at either ages 15 or 17 - Female - Highest IDACI - Sexual minority - White | 2 | 3 | 51.21 | 39.10 | 63.42 | 51.14 | 40.07 | 61.86 | 0.07 | -7.41 | 7.46 |
| 21213 | Adolescent mental distress at either ages 15 or 17 - Male - Middle IDACI - Heterosexual/straight - Asian | 8 | 17 | 46.18 | 34.91 | 56.28 | 46.09 | 37.20 | 54.19 | 0.08 | -6.31 | 6.93 |
| 11114 | No adolescent mental distress at either ages 15 or 17 - Male - Lowest IDACI - Heterosexual/straight - Other (including mixed) | 1 | 7 | 17.69 | 10.92 | 26.10 | 17.60 | 11.62 | 25.26 | 0.09 | -3.50 | 4.54 |
| 21313 | Adolescent mental distress at either ages 15 or 17 - Male - Highest IDACI - Heterosexual/straight - Asian | 15 | 32 | 46.52 | 38.24 | 55.69 | 46.42 | 38.12 | 54.11 | 0.10 | -6.88 | 6.55 |
| 11313 | No adolescent mental distress at either ages 15 or 17 - Male - Highest IDACI - Heterosexual/straight - Asian | 9 | 39 | 22.41 | 16.36 | 29.02 | 22.30 | 16.91 | 28.15 | 0.10 | -4.45 | 4.85 |
| 12113 | No adolescent mental distress at either ages 15 or 17 - Female - Lowest IDACI - Heterosexual/straight - Asian | 1 | 3 | 21.52 | 14.40 | 29.82 | 21.41 | 15.33 | 28.18 | 0.11 | -4.19 | 5.48 |
| 22213 | Adolescent mental distress at either ages 15 or 17 - Female - Middle IDACI - Heterosexual/straight - Asian | 10 | 19 | 52.73 | 42.66 | 62.16 | 52.61 | 43.57 | 60.89 | 0.11 | -6.22 | 7.50 |
| 21112 | Adolescent mental distress at either ages 15 or 17 - Male - Lowest IDACI - Heterosexual/straight - Black | 1 | 1 | 46.80 | 33.84 | 60.28 | 46.67 | 35.12 | 58.98 | 0.13 | -7.53 | 6.99 |
| 21321 | Adolescent mental distress at either ages 15 or 17 - Male - Highest IDACI - Sexual minority - White | 7 | 9 | 70.90 | 60.85 | 80.58 | 70.76 | 61.09 | 79.57 | 0.14 | -5.35 | 5.71 |
| 21314 | Adolescent mental distress at either ages 15 or 17 - Male - Highest IDACI - Heterosexual/straight - Other (including mixed) | 5 | 9 | 46.91 | 35.79 | 59.65 | 46.73 | 35.79 | 58.26 | 0.18 | -6.87 | 6.96 |
| 21214 | Adolescent mental distress at either ages 15 or 17 - Male - Middle IDACI - Heterosexual/straight - Other (including mixed) | 3 | 7 | 46.59 | 35.03 | 59.10 | 46.40 | 35.57 | 57.65 | 0.19 | -6.98 | 7.63 |
| 11214 | No adolescent mental distress at either ages 15 or 17 - Male - Middle IDACI - Heterosexual/straight - Other (including mixed) | 3 | 10 | 22.64 | 14.09 | 32.07 | 22.42 | 15.04 | 31.05 | 0.21 | -4.29 | 5.19 |
| 21221 | Adolescent mental distress at either ages 15 or 17 - Male - Middle IDACI - Sexual minority - White | 10 | 13 | 70.73 | 61.72 | 79.70 | 70.51 | 61.96 | 78.29 | 0.21 | -5.23 | 6.07 |
| 21114 | Adolescent mental distress at either ages 15 or 17 - Male - Lowest IDACI - Heterosexual/straight - Other (including mixed) | 2 | 4 | 39.32 | 29.19 | 52.08 | 39.04 | 28.83 | 50.81 | 0.28 | -6.29 | 6.80 |
| 11111 | No adolescent mental distress at either ages 15 or 17 - Male - Lowest IDACI - Heterosexual/straight - White | 51 | 223 | 22.13 | 18.18 | 26.62 | 21.83 | 17.81 | 26.43 | 0.30 | -3.99 | 4.60 |
| 22124 | Adolescent mental distress at either ages 15 or 17 - Female - Lowest IDACI - Sexual minority - Other (including mixed) | 2 | 2 | 63.59 | 48.91 | 77.70 | 63.28 | 49.96 | 75.96 | 0.32 | -5.84 | 7.64 |
| 11113 | No adolescent mental distress at either ages 15 or 17 - Male - Lowest IDACI - Heterosexual/straight - Asian | 3 | 9 | 17.66 | 11.99 | 24.74 | 17.33 | 12.29 | 23.05 | 0.33 | -3.25 | 4.63 |
| 11121 | No adolescent mental distress at either ages 15 or 17 - Male - Lowest IDACI - Sexual minority - White | 5 | 12 | 37.37 | 26.88 | 49.18 | 37.04 | 28.11 | 46.32 | 0.34 | -5.82 | 7.47 |
| 12214 | No adolescent mental distress at either ages 15 or 17 - Female - Middle IDACI - Heterosexual/straight - Other (including mixed) | 2 | 5 | 27.66 | 17.62 | 38.29 | 27.27 | 18.62 | 37.10 | 0.39 | -5.37 | 6.53 |
| 11212 | No adolescent mental distress at either ages 15 or 17 - Male - Middle IDACI - Heterosexual/straight - Black | 3 | 9 | 28.76 | 18.08 | 40.08 | 28.34 | 19.14 | 37.99 | 0.42 | -4.36 | 6.87 |
| 22221 | Adolescent mental distress at either ages 15 or 17 - Female - Middle IDACI - Sexual minority - White | 16 | 19 | 76.13 | 67.49 | 84.09 | 75.64 | 67.87 | 82.29 | 0.50 | -4.09 | 6.42 |
| 11221 | No adolescent mental distress at either ages 15 or 17 - Male - Middle IDACI - Sexual minority - White | 7 | 12 | 44.99 | 33.47 | 57.38 | 44.32 | 34.71 | 54.32 | 0.68 | -5.02 | 8.82 |
| 22113 | Adolescent mental distress at either ages 15 or 17 - Female - Lowest IDACI - Heterosexual/straight - Asian | 4 | 6 | 45.74 | 35.32 | 57.23 | 45.06 | 36.12 | 53.73 | 0.69 | -6.20 | 8.60 |
| 21312 | Adolescent mental distress at either ages 15 or 17 - Male - Highest IDACI - Heterosexual/straight - Black | 9 | 13 | 55.24 | 43.04 | 68.45 | 54.52 | 44.00 | 65.50 | 0.72 | -6.08 | 8.20 |
| 12121 | No adolescent mental distress at either ages 15 or 17 - Female - Lowest IDACI - Sexual minority - White | 6 | 9 | 44.12 | 33.53 | 56.23 | 43.29 | 33.44 | 53.51 | 0.83 | -5.26 | 8.77 |
| 22314 | Adolescent mental distress at either ages 15 or 17 - Female - Highest IDACI - Heterosexual/straight - Other (including mixed) | 14 | 22 | 54.18 | 43.25 | 65.81 | 53.22 | 42.68 | 63.39 | 0.96 | -5.01 | 8.51 |
| 12211 | No adolescent mental distress at either ages 15 or 17 - Female - Middle IDACI - Heterosexual/straight - White | 47 | 131 | 33.96 | 28.43 | 39.87 | 32.99 | 27.60 | 38.18 | 0.98 | -3.94 | 7.00 |
| 12312 | No adolescent mental distress at either ages 15 or 17 - Female - Highest IDACI - Heterosexual/straight - Black | 7 | 12 | 35.27 | 24.69 | 47.44 | 34.17 | 25.08 | 44.65 | 1.10 | -4.34 | 8.83 |
| 12111 | No adolescent mental distress at either ages 15 or 17 - Female - Lowest IDACI - Heterosexual/straight - White | 47 | 153 | 27.77 | 22.81 | 33.56 | 26.63 | 22.28 | 31.53 | 1.14 | -2.54 | 6.09 |
| 22313 | Adolescent mental distress at either ages 15 or 17 - Female - Highest IDACI - Heterosexual/straight - Asian | 43 | 73 | 54.30 | 46.37 | 62.58 | 52.95 | 44.99 | 60.28 | 1.35 | -3.51 | 7.69 |
| 21211 | Adolescent mental distress at either ages 15 or 17 - Male - Middle IDACI - Heterosexual/straight - White | 85 | 131 | 56.76 | 49.96 | 64.48 | 53.39 | 47.52 | 59.37 | 3.37 | -1.45 | 11.64 |

*Note.* I+M effects = interaction effects and main effects conflated; M effects = main effects only; I effects = differences between intersections and main effects.

## Table A2. Predicted incidence (%) of mental distress at age 25 for individuals who attended university by social strata based on conflated interaction effects and main effects, main effects only, and differences between intersections and main effects, ranked according to the extent to which each interaction effect differs from what is explained by the main effects alone (i.e. the residuals)

| Stratum | Stratum Label | Number of cases | Number of individuals | Predicted incidence (I+M effects) | Low 95% CI predicted incidence (I+M effects) | High 95% CI predicted incidence (I+M effects) | Predicted incidence (M effects) | Low 95% CI predicted incidence (M effects) | High 95% CI predicted incidence (M effects) | Predicted incidence (I effects) | Low 95% CI predicted incidence (I effects) | High 95% CI predicted incidence (I effects) |
| --- | --- | --- | --- | --- | --- | --- | --- | --- | --- | --- | --- | --- |
| 21111 | Adolescent mental distress at either ages 15 or 17 - Male - Lowest IDACI - Heterosexual/straight - White | 94 | 202 | 49.65 | 44.27 | 54.34 | 50.51 | 45.73 | 55.50 | -0.86 | -6.46 | 2.35 |
| 22112 | Adolescent mental distress at either ages 15 or 17 - Female - Lowest IDACI - Heterosexual/straight - Black | 3 | 11 | 53.98 | 44.40 | 63.92 | 54.51 | 45.51 | 63.74 | -0.53 | -5.54 | 3.14 |
| 22314 | Adolescent mental distress at either ages 15 or 17 - Female - Highest IDACI - Heterosexual/straight - Other (including mixed) | 7 | 18 | 53.15 | 43.22 | 62.77 | 53.55 | 44.29 | 62.85 | -0.40 | -5.02 | 3.01 |
| 12314 | No adolescent mental distress at either ages 15 or 17 - Female - Highest IDACI - Heterosexual/straight - Other (including mixed) | 0 | 7 | 33.16 | 24.23 | 42.76 | 33.55 | 25.20 | 42.91 | -0.39 | -4.17 | 2.70 |
| 12113 | No adolescent mental distress at either ages 15 or 17 - Female - Lowest IDACI - Heterosexual/straight - Asian | 4 | 17 | 36.14 | 28.81 | 43.65 | 36.46 | 30.02 | 43.24 | -0.32 | -4.37 | 3.63 |
| 12221 | No adolescent mental distress at either ages 15 or 17 - Female - Middle IDACI - Sexual minority - White | 2 | 7 | 50.18 | 40.22 | 60.47 | 50.50 | 41.01 | 60.06 | -0.32 | -4.82 | 3.30 |
| 22114 | Adolescent mental distress at either ages 15 or 17 - Female - Lowest IDACI - Heterosexual/straight - Other (including mixed) | 10 | 21 | 54.92 | 45.44 | 63.84 | 55.22 | 46.46 | 63.84 | -0.31 | -4.65 | 3.66 |
| 12213 | No adolescent mental distress at either ages 15 or 17 - Female - Middle IDACI - Heterosexual/straight - Asian | 8 | 34 | 32.11 | 25.13 | 38.40 | 32.41 | 26.48 | 38.33 | -0.30 | -4.35 | 2.56 |
| 22211 | Adolescent mental distress at either ages 15 or 17 - Female - Middle IDACI - Heterosexual/straight - White | 100 | 196 | 52.34 | 47.31 | 57.54 | 52.63 | 47.77 | 57.67 | -0.29 | -3.92 | 2.94 |
| 21313 | Adolescent mental distress at either ages 15 or 17 - Male - Highest IDACI - Heterosexual/straight - Asian | 16 | 37 | 48.30 | 41.19 | 55.31 | 48.57 | 42.43 | 54.84 | -0.27 | -4.25 | 3.61 |
| 22221 | Adolescent mental distress at either ages 15 or 17 - Female - Middle IDACI - Sexual minority - White | 8 | 15 | 69.73 | 61.02 | 77.59 | 69.99 | 62.36 | 77.27 | -0.26 | -3.81 | 3.06 |
| 12212 | No adolescent mental distress at either ages 15 or 17 - Female - Middle IDACI - Heterosexual/straight - Black | 2 | 12 | 30.23 | 22.00 | 38.63 | 30.48 | 23.00 | 38.50 | -0.25 | -3.97 | 2.69 |
| 21121 | Adolescent mental distress at either ages 15 or 17 - Male - Lowest IDACI - Sexual minority - White | 15 | 24 | 67.98 | 59.51 | 75.41 | 68.20 | 60.39 | 75.48 | -0.22 | -4.39 | 2.81 |
| 21113 | Adolescent mental distress at either ages 15 or 17 - Male - Lowest IDACI - Heterosexual/straight - Asian | 5 | 12 | 50.04 | 42.56 | 57.73 | 50.26 | 43.41 | 57.49 | -0.22 | -4.85 | 3.55 |
| 11314 | No adolescent mental distress at either ages 15 or 17 - Male - Highest IDACI - Heterosexual/straight - Other (including mixed) | 0 | 7 | 27.76 | 20.11 | 36.65 | 27.97 | 20.58 | 36.52 | -0.21 | -3.66 | 2.88 |
| 22222 | Adolescent mental distress at either ages 15 or 17 - Female - Middle IDACI - Sexual minority - Black | 0 | 2 | 67.52 | 55.38 | 77.79 | 67.72 | 57.11 | 77.49 | -0.20 | -4.52 | 3.51 |
| 12312 | No adolescent mental distress at either ages 15 or 17 - Female - Highest IDACI - Heterosexual/straight - Black | 7 | 25 | 32.70 | 24.66 | 41.30 | 32.88 | 25.22 | 41.67 | -0.19 | -3.88 | 3.23 |
| 21221 | Adolescent mental distress at either ages 15 or 17 - Male - Middle IDACI - Sexual minority - White | 5 | 10 | 64.03 | 54.90 | 73.37 | 64.20 | 55.74 | 72.57 | -0.18 | -4.07 | 3.43 |
| 12214 | No adolescent mental distress at either ages 15 or 17 - Female - Middle IDACI - Heterosexual/straight - Other (including mixed) | 1 | 9 | 30.94 | 22.14 | 39.64 | 31.11 | 23.09 | 39.62 | -0.18 | -3.81 | 3.02 |
| 11311 | No adolescent mental distress at either ages 15 or 17 - Male - Highest IDACI - Heterosexual/straight - White | 8 | 33 | 29.21 | 23.27 | 35.53 | 29.37 | 24.17 | 34.90 | -0.16 | -3.58 | 2.98 |
| 22213 | Adolescent mental distress at either ages 15 or 17 - Female - Middle IDACI - Heterosexual/straight - Asian | 35 | 69 | 52.21 | 45.48 | 58.58 | 52.37 | 45.80 | 57.92 | -0.16 | -4.21 | 3.93 |
| 21112 | Adolescent mental distress at either ages 15 or 17 - Male - Lowest IDACI - Heterosexual/straight - Black | 2 | 4 | 47.83 | 37.21 | 58.74 | 47.96 | 38.46 | 57.68 | -0.13 | -4.53 | 3.82 |
| 21224 | Adolescent mental distress at either ages 15 or 17 - Male - Middle IDACI - Sexual minority - Other (including mixed) | 0 | 1 | 62.32 | 49.51 | 73.73 | 62.43 | 50.64 | 73.22 | -0.12 | -4.77 | 3.44 |
| 11323 | No adolescent mental distress at either ages 15 or 17 - Male - Highest IDACI - Sexual minority - Asian | 0 | 2 | 46.37 | 35.25 | 56.97 | 46.48 | 36.30 | 56.71 | -0.11 | -4.41 | 3.94 |
| 21213 | Adolescent mental distress at either ages 15 or 17 - Male - Middle IDACI - Heterosexual/straight - Asian | 20 | 44 | 45.69 | 38.75 | 52.86 | 45.80 | 39.22 | 52.22 | -0.10 | -4.48 | 3.94 |
| 11224 | No adolescent mental distress at either ages 15 or 17 - Male - Middle IDACI - Sexual minority - Other (including mixed) | 0 | 1 | 42.14 | 30.47 | 54.74 | 42.23 | 30.76 | 54.57 | -0.10 | -3.84 | 4.21 |
| 11113 | No adolescent mental distress at either ages 15 or 17 - Male - Lowest IDACI - Heterosexual/straight - Asian | 9 | 33 | 30.49 | 24.00 | 37.35 | 30.59 | 24.72 | 36.77 | -0.10 | -3.72 | 3.40 |
| 12311 | No adolescent mental distress at either ages 15 or 17 - Female - Highest IDACI - Heterosexual/straight - White | 11 | 33 | 35.03 | 28.46 | 41.84 | 35.12 | 29.22 | 41.37 | -0.09 | -3.51 | 3.22 |
| 12222 | No adolescent mental distress at either ages 15 or 17 - Female - Middle IDACI - Sexual minority - Black | 0 | 1 | 47.88 | 34.64 | 59.89 | 47.97 | 35.20 | 59.93 | -0.09 | -4.23 | 3.63 |
| 21321 | Adolescent mental distress at either ages 15 or 17 - Male - Highest IDACI - Sexual minority - White | 3 | 5 | 66.61 | 57.17 | 75.57 | 66.69 | 57.69 | 74.84 | -0.09 | -3.70 | 3.23 |
| 22323 | Adolescent mental distress at either ages 15 or 17 - Female - Highest IDACI - Sexual minority - Asian | 1 | 2 | 71.94 | 62.16 | 80.00 | 72.03 | 63.19 | 79.82 | -0.09 | -4.05 | 3.49 |
| 11211 | No adolescent mental distress at either ages 15 or 17 - Male - Middle IDACI - Heterosexual/straight - White | 36 | 138 | 27.00 | 22.80 | 31.72 | 27.08 | 23.31 | 31.08 | -0.08 | -3.01 | 3.06 |
| 21322 | Adolescent mental distress at either ages 15 or 17 - Male - Highest IDACI - Sexual minority - Black | 0 | 1 | 64.26 | 51.74 | 74.80 | 64.33 | 53.26 | 74.28 | -0.08 | -4.25 | 3.98 |
| 12321 | No adolescent mental distress at either ages 15 or 17 - Female - Highest IDACI - Sexual minority - White | 2 | 4 | 53.18 | 42.19 | 64.27 | 53.26 | 42.90 | 63.53 | -0.08 | -4.80 | 4.04 |
| 22111 | Adolescent mental distress at either ages 15 or 17 - Female - Lowest IDACI - Heterosexual/straight - White | 222 | 390 | 57.02 | 53.22 | 61.04 | 57.07 | 52.96 | 61.62 | -0.05 | -3.63 | 3.21 |
| 21223 | Adolescent mental distress at either ages 15 or 17 - Male - Middle IDACI - Sexual minority - Asian | 1 | 2 | 63.89 | 52.44 | 73.47 | 63.93 | 53.39 | 73.25 | -0.04 | -3.90 | 3.40 |
| 12224 | No adolescent mental distress at either ages 15 or 17 - Female - Middle IDACI - Sexual minority - Other (including mixed) | 0 | 1 | 48.64 | 35.91 | 61.62 | 48.68 | 36.96 | 61.49 | -0.04 | -4.49 | 4.08 |
| 21323 | Adolescent mental distress at either ages 15 or 17 - Male - Highest IDACI - Sexual minority - Asian | 2 | 3 | 66.42 | 55.84 | 75.28 | 66.45 | 56.28 | 75.46 | -0.03 | -3.70 | 3.74 |
| 21314 | Adolescent mental distress at either ages 15 or 17 - Male - Highest IDACI - Heterosexual/straight - Other (including mixed) | 6 | 12 | 46.98 | 36.91 | 57.04 | 47.01 | 37.26 | 56.86 | -0.03 | -4.38 | 3.89 |
| 11321 | No adolescent mental distress at either ages 15 or 17 - Male - Highest IDACI - Sexual minority - White | 2 | 3 | 46.70 | 35.89 | 57.37 | 46.73 | 36.91 | 56.56 | -0.03 | -4.18 | 4.22 |
| 11313 | No adolescent mental distress at either ages 15 or 17 - Male - Highest IDACI - Heterosexual/straight - Asian | 14 | 49 | 29.13 | 23.78 | 34.85 | 29.16 | 24.31 | 34.36 | -0.02 | -2.97 | 3.27 |
| 11312 | No adolescent mental distress at either ages 15 or 17 - Male - Highest IDACI - Heterosexual/straight - Black | 6 | 23 | 27.35 | 20.09 | 35.53 | 27.37 | 20.49 | 35.26 | -0.02 | -2.79 | 3.26 |
| 12121 | No adolescent mental distress at either ages 15 or 17 - Female - Lowest IDACI - Sexual minority - White | 3 | 5 | 54.95 | 44.59 | 64.57 | 54.94 | 45.59 | 64.26 | 0.01 | -3.50 | 3.79 |
| 12111 | No adolescent mental distress at either ages 15 or 17 - Female - Lowest IDACI - Heterosexual/straight - White | 74 | 202 | 36.67 | 32.23 | 41.24 | 36.65 | 32.38 | 41.30 | 0.02 | -3.71 | 3.25 |
| 21222 | Adolescent mental distress at either ages 15 or 17 - Male - Middle IDACI - Sexual minority - Black | 1 | 1 | 61.78 | 48.43 | 73.09 | 61.76 | 49.30 | 72.36 | 0.02 | -3.85 | 3.65 |
| 21114 | Adolescent mental distress at either ages 15 or 17 - Male - Lowest IDACI - Heterosexual/straight - Other (including mixed) | 9 | 18 | 48.71 | 38.51 | 59.33 | 48.68 | 39.02 | 58.36 | 0.03 | -4.63 | 4.80 |
| 21324 | Adolescent mental distress at either ages 15 or 17 - Male - Highest IDACI - Sexual minority - Other (including mixed) | 1 | 1 | 65.02 | 53.10 | 76.05 | 64.98 | 53.50 | 75.58 | 0.04 | -3.09 | 3.99 |
| 21212 | Adolescent mental distress at either ages 15 or 17 - Male - Middle IDACI - Heterosexual/straight - Black | 4 | 10 | 43.59 | 34.39 | 53.36 | 43.54 | 34.48 | 53.33 | 0.05 | -3.68 | 4.34 |
| 22124 | Adolescent mental distress at either ages 15 or 17 - Female - Lowest IDACI - Sexual minority - Other (including mixed) | 2 | 2 | 72.12 | 61.55 | 81.29 | 72.06 | 61.61 | 80.77 | 0.07 | -3.12 | 3.43 |
| 12324 | No adolescent mental distress at either ages 15 or 17 - Female - Highest IDACI - Sexual minority - Other (including mixed) | 1 | 1 | 51.50 | 38.92 | 64.64 | 51.43 | 39.27 | 64.06 | 0.07 | -4.02 | 4.44 |
| 11121 | No adolescent mental distress at either ages 15 or 17 - Male - Lowest IDACI - Sexual minority - White | 6 | 11 | 48.47 | 39.00 | 58.21 | 48.40 | 39.05 | 57.36 | 0.07 | -3.65 | 4.43 |
| 11221 | No adolescent mental distress at either ages 15 or 17 - Male - Middle IDACI - Sexual minority - White | 3 | 6 | 44.05 | 34.38 | 53.89 | 43.97 | 35.04 | 53.65 | 0.08 | -3.81 | 4.32 |
| 11111 | No adolescent mental distress at either ages 15 or 17 - Male - Lowest IDACI - Heterosexual/straight - White | 76 | 242 | 30.84 | 26.73 | 35.15 | 30.76 | 26.99 | 34.89 | 0.08 | -2.94 | 3.50 |
| 22224 | Adolescent mental distress at either ages 15 or 17 - Female - Middle IDACI - Sexual minority - Other (including mixed) | 2 | 2 | 68.42 | 57.48 | 78.37 | 68.34 | 57.40 | 78.30 | 0.08 | -3.10 | 3.77 |
| 22321 | Adolescent mental distress at either ages 15 or 17 - Female - Highest IDACI - Sexual minority - White | 5 | 6 | 72.35 | 63.72 | 79.97 | 72.25 | 64.35 | 79.49 | 0.10 | -3.01 | 3.54 |
| 21123 | Adolescent mental distress at either ages 15 or 17 - Male - Lowest IDACI - Sexual minority - Asian | 1 | 1 | 68.02 | 57.83 | 77.25 | 67.91 | 58.17 | 76.63 | 0.10 | -3.31 | 3.81 |
| 22214 | Adolescent mental distress at either ages 15 or 17 - Female - Middle IDACI - Heterosexual/straight - Other (including mixed) | 10 | 17 | 50.89 | 41.14 | 60.50 | 50.79 | 41.96 | 59.55 | 0.11 | -4.18 | 4.54 |
| 21312 | Adolescent mental distress at either ages 15 or 17 - Male - Highest IDACI - Heterosexual/straight - Black | 6 | 11 | 46.43 | 36.62 | 56.37 | 46.28 | 37.64 | 55.45 | 0.14 | -3.64 | 4.80 |
| 12323 | No adolescent mental distress at either ages 15 or 17 - Female - Highest IDACI - Sexual minority - Asian | 2 | 2 | 53.15 | 41.78 | 64.62 | 53.01 | 42.68 | 64.06 | 0.15 | -3.47 | 4.30 |
| 22322 | Adolescent mental distress at either ages 15 or 17 - Female - Highest IDACI - Sexual minority - Black | 2 | 2 | 70.26 | 59.40 | 79.55 | 70.09 | 59.80 | 79.09 | 0.17 | -3.02 | 3.58 |
| 21311 | Adolescent mental distress at either ages 15 or 17 - Male - Highest IDACI - Heterosexual/straight - White | 12 | 22 | 48.99 | 41.40 | 56.41 | 48.82 | 42.00 | 55.24 | 0.17 | -3.59 | 4.96 |
| 22312 | Adolescent mental distress at either ages 15 or 17 - Female - Highest IDACI - Heterosexual/straight - Black | 26 | 47 | 53.02 | 44.02 | 61.20 | 52.84 | 44.46 | 61.18 | 0.18 | -3.71 | 4.43 |
| 22324 | Adolescent mental distress at either ages 15 or 17 - Female - Highest IDACI - Sexual minority - Other (including mixed) | 3 | 3 | 70.87 | 59.75 | 80.45 | 70.68 | 60.27 | 80.07 | 0.19 | -2.82 | 3.75 |
| 11112 | No adolescent mental distress at either ages 15 or 17 - Male - Lowest IDACI - Heterosexual/straight - Black | 2 | 3 | 28.96 | 21.58 | 38.40 | 28.75 | 21.45 | 37.37 | 0.21 | -3.24 | 3.77 |
| 11114 | No adolescent mental distress at either ages 15 or 17 - Male - Lowest IDACI - Heterosexual/straight - Other (including mixed) | 4 | 9 | 29.55 | 21.65 | 38.26 | 29.33 | 21.84 | 37.13 | 0.21 | -2.87 | 3.99 |
| 11213 | No adolescent mental distress at either ages 15 or 17 - Male - Middle IDACI - Heterosexual/straight - Asian | 18 | 58 | 27.13 | 21.78 | 33.12 | 26.91 | 22.11 | 32.33 | 0.22 | -2.55 | 3.66 |
| 22313 | Adolescent mental distress at either ages 15 or 17 - Female - Highest IDACI - Heterosexual/straight - Asian | 51 | 89 | 55.37 | 49.09 | 61.52 | 55.14 | 48.90 | 60.78 | 0.23 | -3.54 | 4.58 |
| 12313 | No adolescent mental distress at either ages 15 or 17 - Female - Highest IDACI - Heterosexual/straight - Asian | 19 | 51 | 35.13 | 28.99 | 41.87 | 34.89 | 29.10 | 40.98 | 0.24 | -2.98 | 4.28 |
| 22212 | Adolescent mental distress at either ages 15 or 17 - Female - Middle IDACI - Heterosexual/straight - Black | 13 | 23 | 50.31 | 40.80 | 59.88 | 50.06 | 41.33 | 58.91 | 0.25 | -3.51 | 4.83 |
| 11214 | No adolescent mental distress at either ages 15 or 17 - Male - Middle IDACI - Heterosexual/straight - Other (including mixed) | 5 | 11 | 26.03 | 18.40 | 34.18 | 25.78 | 18.80 | 33.33 | 0.26 | -2.73 | 4.01 |
| 11223 | No adolescent mental distress at either ages 15 or 17 - Male - Middle IDACI - Sexual minority - Asian | 3 | 4 | 44.02 | 32.76 | 56.30 | 43.75 | 33.08 | 54.55 | 0.28 | -3.46 | 4.92 |
| 12114 | No adolescent mental distress at either ages 15 or 17 - Female - Lowest IDACI - Heterosexual/straight - Other (including mixed) | 6 | 11 | 35.40 | 26.22 | 44.52 | 35.05 | 26.66 | 43.73 | 0.35 | -2.81 | 4.61 |
| 21214 | Adolescent mental distress at either ages 15 or 17 - Male - Middle IDACI - Heterosexual/straight - Other (including mixed) | 5 | 8 | 44.60 | 34.80 | 54.50 | 44.26 | 34.99 | 54.05 | 0.35 | -3.31 | 5.13 |
| 21211 | Adolescent mental distress at either ages 15 or 17 - Male - Middle IDACI - Heterosexual/straight - White | 42 | 85 | 46.41 | 40.83 | 52.38 | 46.04 | 40.95 | 51.53 | 0.37 | -3.02 | 4.66 |
| 11212 | No adolescent mental distress at either ages 15 or 17 - Male - Middle IDACI - Heterosexual/straight - Black | 5 | 7 | 25.58 | 18.36 | 33.68 | 25.21 | 18.43 | 32.73 | 0.37 | -2.40 | 4.64 |
| 22121 | Adolescent mental distress at either ages 15 or 17 - Female - Lowest IDACI - Sexual minority - White | 33 | 39 | 74.03 | 66.96 | 80.76 | 73.62 | 66.34 | 80.05 | 0.42 | -2.17 | 3.97 |
| 22311 | Adolescent mental distress at either ages 15 or 17 - Female - Highest IDACI - Heterosexual/straight - White | 37 | 59 | 55.99 | 49.49 | 62.71 | 55.39 | 49.25 | 61.31 | 0.60 | -2.55 | 5.31 |
| 12211 | No adolescent mental distress at either ages 15 or 17 - Female - Middle IDACI - Heterosexual/straight - White | 41 | 110 | 33.23 | 28.17 | 38.65 | 32.61 | 27.91 | 37.50 | 0.62 | -2.18 | 4.62 |
| 22113 | Adolescent mental distress at either ages 15 or 17 - Female - Lowest IDACI - Heterosexual/straight - Asian | 26 | 37 | 57.55 | 50.97 | 65.62 | 56.80 | 50.77 | 63.28 | 0.75 | -2.70 | 5.85 |

*Note.* I+M effects = interaction effects and main effects conflated; M effects = main effects only; I effects = differences between intersections and main effects.

## Table A3. Predicted incidence (%) of chronic mental illness at age 25 for individuals who did not attend university by social strata based on conflated interaction effects and main effects, main effects only, and differences between intersections and main effects, ranked according to the extent to which each interaction effect differs from what is explained by the main effects alone (i.e. the residuals)

| Stratum | Stratum Label | Number of cases | Number of individuals | Predicted incidence (I+M effects) | Low 95% CI predicted incidence (I+M effects) | High 95% CI predicted incidence (I+M effects) | Predicted incidence (M effects) | Low 95% CI predicted incidence (M effects) | High 95% CI predicted incidence (M effects) | Predicted incidence (I effects) | Low 95% CI predicted incidence (I effects) | High 95% CI predicted incidence (I effects) |
| --- | --- | --- | --- | --- | --- | --- | --- | --- | --- | --- | --- | --- |
| 22321 | Adolescent mental distress at either ages 15 or 17 - Female - Highest IDACI - Sexual minority - White | 2 | 11 | 41.65 | 27.97 | 54.04 | 42.89 | 30.77 | 54.71 | -1.24 | -10.63 | 5.02 |
| 22311 | Adolescent mental distress at either ages 15 or 17 - Female - Highest IDACI - Heterosexual/straight - White | 18 | 136 | 15.83 | 10.88 | 20.58 | 16.65 | 12.01 | 21.91 | -0.82 | -5.52 | 2.17 |
| 21321 | Adolescent mental distress at either ages 15 or 17 - Male - Highest IDACI - Sexual minority - White | 2 | 9 | 34.34 | 21.89 | 47.25 | 34.96 | 23.74 | 46.88 | -0.62 | -8.33 | 5.95 |
| 21111 | Adolescent mental distress at either ages 15 or 17 - Male - Lowest IDACI - Heterosexual/straight - White | 10 | 148 | 8.29 | 5.49 | 11.46 | 8.61 | 5.93 | 11.97 | -0.32 | -2.91 | 1.58 |
| 22121 | Adolescent mental distress at either ages 15 or 17 - Female - Lowest IDACI - Sexual minority - White | 5 | 17 | 32.99 | 22.20 | 45.72 | 33.24 | 23.12 | 44.25 | -0.25 | -7.99 | 6.68 |
| 22124 | Adolescent mental distress at either ages 15 or 17 - Female - Lowest IDACI - Sexual minority - Other (including mixed) | 0 | 2 | 23.26 | 8.91 | 40.83 | 23.48 | 9.85 | 40.19 | -0.21 | -6.08 | 4.95 |
| 11221 | No adolescent mental distress at either ages 15 or 17 - Male - Middle IDACI - Sexual minority - White | 1 | 12 | 16.60 | 9.92 | 25.16 | 16.80 | 10.49 | 25.12 | -0.21 | -4.97 | 3.98 |
| 11211 | No adolescent mental distress at either ages 15 or 17 - Male - Middle IDACI - Heterosexual/straight - White | 8 | 199 | 4.90 | 3.10 | 6.93 | 5.04 | 3.39 | 7.04 | -0.15 | -1.64 | 1.08 |
| 22324 | Adolescent mental distress at either ages 15 or 17 - Female - Highest IDACI - Sexual minority - Other (including mixed) | 0 | 1 | 31.25 | 14.21 | 51.03 | 31.39 | 14.81 | 49.01 | -0.14 | -6.93 | 7.03 |
| 12221 | No adolescent mental distress at either ages 15 or 17 - Female - Middle IDACI - Sexual minority - White | 2 | 12 | 21.94 | 13.12 | 32.68 | 22.07 | 13.94 | 31.71 | -0.13 | -5.54 | 4.86 |
| 22114 | Adolescent mental distress at either ages 15 or 17 - Female - Lowest IDACI - Heterosexual/straight - Other (including mixed) | 0 | 15 | 7.49 | 2.93 | 14.20 | 7.61 | 2.99 | 14.17 | -0.12 | -2.75 | 2.40 |
| 12211 | No adolescent mental distress at either ages 15 or 17 - Female - Middle IDACI - Heterosexual/straight - White | 8 | 132 | 6.84 | 4.41 | 9.60 | 6.93 | 4.77 | 9.66 | -0.10 | -2.04 | 1.53 |
| 11111 | No adolescent mental distress at either ages 15 or 17 - Male - Lowest IDACI - Heterosexual/straight - White | 6 | 226 | 3.46 | 2.17 | 5.13 | 3.55 | 2.38 | 5.26 | -0.10 | -1.39 | 0.86 |
| 21314 | Adolescent mental distress at either ages 15 or 17 - Male - Highest IDACI - Heterosexual/straight - Other (including mixed) | 0 | 9 | 8.11 | 3.06 | 15.28 | 8.17 | 3.37 | 15.04 | -0.06 | -2.46 | 2.74 |
| 22322 | Adolescent mental distress at either ages 15 or 17 - Female - Highest IDACI - Sexual minority - Black | 0 | 2 | 17.32 | 4.82 | 34.91 | 17.37 | 4.92 | 34.32 | -0.06 | -4.58 | 5.32 |
| 22313 | Adolescent mental distress at either ages 15 or 17 - Female - Highest IDACI - Heterosexual/straight - Asian | 4 | 77 | 5.96 | 2.91 | 10.00 | 6.02 | 2.93 | 10.39 | -0.06 | -2.27 | 1.78 |
| 11121 | No adolescent mental distress at either ages 15 or 17 - Male - Lowest IDACI - Sexual minority - White | 1 | 12 | 12.26 | 6.82 | 19.91 | 12.32 | 7.37 | 19.11 | -0.05 | -3.72 | 3.64 |
| 12214 | No adolescent mental distress at either ages 15 or 17 - Female - Middle IDACI - Heterosexual/straight - Other (including mixed) | 0 | 5 | 4.44 | 1.52 | 8.85 | 4.48 | 1.79 | 8.77 | -0.03 | -1.44 | 1.49 |
| 12312 | No adolescent mental distress at either ages 15 or 17 - Female - Highest IDACI - Heterosexual/straight - Black | 0 | 12 | 2.18 | 0.54 | 4.96 | 2.20 | 0.58 | 4.95 | -0.02 | -0.82 | 0.69 |
| 11214 | No adolescent mental distress at either ages 15 or 17 - Male - Middle IDACI - Heterosexual/straight - Other (including mixed) | 0 | 10 | 3.22 | 1.21 | 6.36 | 3.23 | 1.24 | 6.15 | -0.02 | -1.13 | 1.00 |
| 22111 | Adolescent mental distress at either ages 15 or 17 - Female - Lowest IDACI - Heterosexual/straight - White | 28 | 240 | 11.62 | 8.79 | 15.09 | 11.63 | 8.66 | 15.39 | -0.01 | -2.85 | 2.68 |
| 11314 | No adolescent mental distress at either ages 15 or 17 - Male - Highest IDACI - Heterosexual/straight - Other (including mixed) | 0 | 12 | 3.37 | 1.22 | 6.92 | 3.38 | 1.37 | 6.65 | -0.01 | -1.04 | 1.19 |
| 21313 | Adolescent mental distress at either ages 15 or 17 - Male - Highest IDACI - Heterosexual/straight - Asian | 1 | 35 | 4.37 | 1.94 | 7.71 | 4.37 | 2.00 | 7.60 | -0.01 | -1.23 | 1.31 |
| 21214 | Adolescent mental distress at either ages 15 or 17 - Male - Middle IDACI - Heterosexual/straight - Other (including mixed) | 0 | 7 | 7.84 | 2.73 | 14.75 | 7.85 | 3.13 | 14.37 | -0.01 | -1.95 | 2.63 |
| 22214 | Adolescent mental distress at either ages 15 or 17 - Female - Middle IDACI - Heterosexual/straight - Other (including mixed) | 1 | 13 | 10.60 | 4.26 | 19.16 | 10.60 | 4.46 | 19.17 | -0.01 | -3.00 | 2.96 |
| 11213 | No adolescent mental distress at either ages 15 or 17 - Male - Middle IDACI - Heterosexual/straight - Asian | 0 | 17 | 1.69 | 0.69 | 3.29 | 1.69 | 0.75 | 3.16 | 0.00 | -0.57 | 0.60 |
| 11212 | No adolescent mental distress at either ages 15 or 17 - Male - Middle IDACI - Heterosexual/straight - Black | 0 | 9 | 1.52 | 0.38 | 3.53 | 1.52 | 0.40 | 3.56 | 0.00 | -0.56 | 0.62 |
| 22112 | Adolescent mental distress at either ages 15 or 17 - Female - Lowest IDACI - Heterosexual/straight - Black | 0 | 4 | 3.68 | 0.90 | 8.31 | 3.68 | 0.92 | 8.30 | 0.00 | -1.22 | 1.31 |
| 12213 | No adolescent mental distress at either ages 15 or 17 - Female - Middle IDACI - Heterosexual/straight - Asian | 0 | 17 | 2.37 | 0.96 | 4.55 | 2.36 | 1.03 | 4.36 | 0.00 | -0.73 | 0.84 |
| 21112 | Adolescent mental distress at either ages 15 or 17 - Male - Lowest IDACI - Heterosexual/straight - Black | 0 | 1 | 2.67 | 0.61 | 6.41 | 2.66 | 0.68 | 6.09 | 0.01 | -0.89 | 0.95 |
| 12323 | No adolescent mental distress at either ages 15 or 17 - Female - Highest IDACI - Sexual minority - Asian | 0 | 3 | 8.82 | 3.53 | 18.22 | 8.81 | 3.84 | 17.73 | 0.01 | -2.50 | 2.89 |
| 11312 | No adolescent mental distress at either ages 15 or 17 - Male - Highest IDACI - Heterosexual/straight - Black | 0 | 15 | 1.59 | 0.40 | 3.82 | 1.58 | 0.44 | 3.54 | 0.01 | -0.47 | 0.54 |
| 21113 | Adolescent mental distress at either ages 15 or 17 - Male - Lowest IDACI - Heterosexual/straight - Asian | 0 | 11 | 2.97 | 1.17 | 5.79 | 2.96 | 1.32 | 5.35 | 0.01 | -0.99 | 1.07 |
| 12113 | No adolescent mental distress at either ages 15 or 17 - Female - Lowest IDACI - Heterosexual/straight - Asian | 0 | 4 | 1.68 | 0.64 | 3.40 | 1.66 | 0.69 | 3.27 | 0.02 | -0.49 | 0.58 |
| 11222 | No adolescent mental distress at either ages 15 or 17 - Male - Middle IDACI - Sexual minority - Black | 0 | 1 | 5.53 | 1.31 | 13.55 | 5.52 | 1.39 | 13.38 | 0.02 | -1.58 | 1.98 |
| 12111 | No adolescent mental distress at either ages 15 or 17 - Female - Lowest IDACI - Heterosexual/straight - White | 8 | 154 | 4.93 | 3.20 | 7.30 | 4.92 | 3.32 | 7.00 | 0.02 | -1.40 | 1.45 |
| 22212 | Adolescent mental distress at either ages 15 or 17 - Female - Middle IDACI - Heterosexual/straight - Black | 0 | 5 | 5.22 | 1.36 | 11.98 | 5.20 | 1.42 | 11.73 | 0.02 | -1.57 | 1.55 |
| 11112 | No adolescent mental distress at either ages 15 or 17 - Male - Lowest IDACI - Heterosexual/straight - Black | 0 | 2 | 1.08 | 0.25 | 2.64 | 1.06 | 0.25 | 2.52 | 0.02 | -0.33 | 0.47 |
| 11223 | No adolescent mental distress at either ages 15 or 17 - Male - Middle IDACI - Sexual minority - Asian | 0 | 1 | 6.19 | 2.46 | 13.10 | 6.16 | 2.62 | 12.25 | 0.03 | -1.77 | 1.84 |
| 12212 | No adolescent mental distress at either ages 15 or 17 - Female - Middle IDACI - Heterosexual/straight - Black | 0 | 3 | 2.15 | 0.51 | 5.12 | 2.12 | 0.55 | 5.05 | 0.03 | -0.60 | 0.84 |
| 11313 | No adolescent mental distress at either ages 15 or 17 - Male - Highest IDACI - Heterosexual/straight - Asian | 1 | 40 | 1.80 | 0.75 | 3.47 | 1.76 | 0.78 | 3.17 | 0.03 | -0.50 | 0.69 |
| 21212 | Adolescent mental distress at either ages 15 or 17 - Male - Middle IDACI - Heterosexual/straight - Black | 0 | 5 | 3.82 | 0.94 | 9.19 | 3.78 | 0.99 | 8.71 | 0.04 | -1.07 | 1.38 |
| 22213 | Adolescent mental distress at either ages 15 or 17 - Female - Middle IDACI - Heterosexual/straight - Asian | 1 | 22 | 5.83 | 2.54 | 10.48 | 5.79 | 2.77 | 9.99 | 0.04 | -1.60 | 2.27 |
| 12114 | No adolescent mental distress at either ages 15 or 17 - Female - Lowest IDACI - Heterosexual/straight - Other (including mixed) | 0 | 1 | 3.19 | 1.17 | 6.36 | 3.15 | 1.25 | 6.17 | 0.04 | -1.06 | 1.38 |
| 11113 | No adolescent mental distress at either ages 15 or 17 - Male - Lowest IDACI - Heterosexual/straight - Asian | 1 | 9 | 1.23 | 0.50 | 2.59 | 1.18 | 0.52 | 2.25 | 0.05 | -0.31 | 0.61 |
| 21123 | Adolescent mental distress at either ages 15 or 17 - Male - Lowest IDACI - Sexual minority - Asian | 0 | 1 | 10.42 | 4.36 | 20.68 | 10.37 | 4.49 | 19.43 | 0.05 | -2.84 | 3.37 |
| 11323 | No adolescent mental distress at either ages 15 or 17 - Male - Highest IDACI - Sexual minority - Asian | 0 | 1 | 6.47 | 2.74 | 13.16 | 6.42 | 2.70 | 12.34 | 0.05 | -2.01 | 1.87 |
| 11114 | No adolescent mental distress at either ages 15 or 17 - Male - Lowest IDACI - Heterosexual/straight - Other (including mixed) | 1 | 7 | 2.33 | 0.82 | 4.80 | 2.27 | 0.89 | 4.61 | 0.06 | -0.64 | 1.01 |
| 12222 | No adolescent mental distress at either ages 15 or 17 - Female - Middle IDACI - Sexual minority - Black | 0 | 1 | 7.62 | 1.85 | 17.97 | 7.56 | 1.88 | 18.19 | 0.06 | -1.92 | 2.79 |
| 12313 | No adolescent mental distress at either ages 15 or 17 - Female - Highest IDACI - Heterosexual/straight - Asian | 2 | 42 | 2.53 | 1.13 | 4.89 | 2.47 | 1.12 | 4.50 | 0.07 | -0.69 | 1.22 |
| 21213 | Adolescent mental distress at either ages 15 or 17 - Male - Middle IDACI - Heterosexual/straight - Asian | 1 | 18 | 4.28 | 1.87 | 7.89 | 4.20 | 1.92 | 7.52 | 0.07 | -1.25 | 1.66 |
| 12314 | No adolescent mental distress at either ages 15 or 17 - Female - Highest IDACI - Heterosexual/straight - Other (including mixed) | 1 | 11 | 4.75 | 1.85 | 9.52 | 4.68 | 1.87 | 9.24 | 0.08 | -1.42 | 1.84 |
| 11311 | No adolescent mental distress at either ages 15 or 17 - Male - Highest IDACI - Heterosexual/straight - White | 7 | 113 | 5.38 | 3.31 | 8.17 | 5.30 | 3.39 | 7.95 | 0.08 | -1.35 | 2.00 |
| 22312 | Adolescent mental distress at either ages 15 or 17 - Female - Highest IDACI - Heterosexual/straight - Black | 2 | 26 | 5.46 | 1.52 | 12.32 | 5.38 | 1.53 | 11.40 | 0.08 | -1.48 | 2.29 |
| 21114 | Adolescent mental distress at either ages 15 or 17 - Male - Lowest IDACI - Heterosexual/straight - Other (including mixed) | 0 | 4 | 5.66 | 2.00 | 11.30 | 5.58 | 2.13 | 10.61 | 0.08 | -1.66 | 2.27 |
| 21312 | Adolescent mental distress at either ages 15 or 17 - Male - Highest IDACI - Heterosexual/straight - Black | 1 | 15 | 4.01 | 1.02 | 8.91 | 3.92 | 1.08 | 8.49 | 0.09 | -0.99 | 1.89 |
| 22113 | Adolescent mental distress at either ages 15 or 17 - Female - Lowest IDACI - Heterosexual/straight - Asian | 1 | 6 | 4.24 | 1.80 | 8.27 | 4.10 | 1.83 | 7.40 | 0.14 | -0.96 | 2.14 |
| 22221 | Adolescent mental distress at either ages 15 or 17 - Female - Middle IDACI - Sexual minority - White | 8 | 19 | 41.91 | 29.01 | 54.36 | 41.73 | 29.83 | 52.73 | 0.18 | -7.09 | 8.02 |
| 12311 | No adolescent mental distress at either ages 15 or 17 - Female - Highest IDACI - Heterosexual/straight - White | 8 | 82 | 7.53 | 4.85 | 11.60 | 7.28 | 4.64 | 10.59 | 0.25 | -1.67 | 3.16 |
| 12321 | No adolescent mental distress at either ages 15 or 17 - Female - Highest IDACI - Sexual minority - White | 1 | 3 | 23.20 | 13.06 | 36.17 | 22.95 | 14.19 | 34.30 | 0.25 | -4.88 | 6.25 |
| 21311 | Adolescent mental distress at either ages 15 or 17 - Male - Highest IDACI - Heterosexual/straight - White | 11 | 75 | 12.80 | 8.60 | 18.46 | 12.51 | 8.24 | 17.14 | 0.29 | -2.99 | 4.42 |
| 22222 | Adolescent mental distress at either ages 15 or 17 - Female - Middle IDACI - Sexual minority - Black | 1 | 1 | 17.19 | 4.75 | 36.55 | 16.82 | 4.71 | 35.22 | 0.36 | -3.41 | 5.77 |
| 11321 | No adolescent mental distress at either ages 15 or 17 - Male - Highest IDACI - Sexual minority - White | 1 | 3 | 17.93 | 10.13 | 29.87 | 17.53 | 10.64 | 26.76 | 0.39 | -4.05 | 6.67 |
| 22211 | Adolescent mental distress at either ages 15 or 17 - Female - Middle IDACI - Heterosexual/straight - White | 41 | 238 | 16.44 | 12.77 | 20.81 | 15.97 | 11.98 | 20.21 | 0.48 | -2.46 | 4.71 |
| 21211 | Adolescent mental distress at either ages 15 or 17 - Male - Middle IDACI - Heterosexual/straight - White | 20 | 132 | 12.66 | 8.93 | 17.40 | 11.97 | 8.31 | 16.23 | 0.69 | -1.79 | 5.00 |
| 12121 | No adolescent mental distress at either ages 15 or 17 - Female - Lowest IDACI - Sexual minority - White | 4 | 10 | 17.20 | 10.11 | 28.48 | 16.47 | 10.04 | 25.43 | 0.72 | -2.75 | 7.43 |
| 21221 | Adolescent mental distress at either ages 15 or 17 - Male - Middle IDACI - Sexual minority - White | 6 | 13 | 34.66 | 23.87 | 46.75 | 33.87 | 23.68 | 45.30 | 0.79 | -5.03 | 9.38 |
| 22314 | Adolescent mental distress at either ages 15 or 17 - Female - Highest IDACI - Heterosexual/straight - Other (including mixed) | 6 | 22 | 11.91 | 5.11 | 21.11 | 11.03 | 4.67 | 19.54 | 0.89 | -1.67 | 6.52 |
| 21121 | Adolescent mental distress at either ages 15 or 17 - Male - Lowest IDACI - Sexual minority - White | 6 | 13 | 27.43 | 17.71 | 39.57 | 26.26 | 17.41 | 36.68 | 1.17 | -3.78 | 9.82 |

*Note.* I+M effects = interaction effects and main effects conflated; M effects = main effects only; I effects = differences between intersections and main effects.

## Table A4. Predicted incidence (%) of chronic mental illness at age 25 for individuals who attended university by social strata based on conflated interaction effects and main effects, main effects only, and differences between intersections and main effects, ranked according to the extent to which each interaction effect differs from what is explained by the main effects alone (i.e. the residuals)

| Stratum | Stratum Label | Number of cases | Number of individuals | Predicted incidence (I+M effects) | Low 95% CI predicted incidence (I+M effects) | High 95% CI predicted incidence (I+M effects) | Predicted incidence (M effects) | Low 95% CI predicted incidence (M effects) | High 95% CI predicted incidence (M effects) | Predicted incidence (I effects) | Low 95% CI predicted incidence (I effects) | High 95% CI predicted incidence (I effects) |
| --- | --- | --- | --- | --- | --- | --- | --- | --- | --- | --- | --- | --- |
| 22221 | Adolescent mental distress at either ages 15 or 17 - Female - Middle IDACI - Sexual minority - White | 2 | 15 | 26.50 | 16.09 | 37.28 | 27.45 | 18.34 | 37.75 | -0.95 | -8.17 | 4.62 |
| 22311 | Adolescent mental distress at either ages 15 or 17 - Female - Highest IDACI - Heterosexual/straight - White | 4 | 59 | 11.25 | 6.55 | 16.80 | 11.74 | 7.16 | 17.30 | -0.49 | -4.22 | 2.45 |
| 12221 | No adolescent mental distress at either ages 15 or 17 - Female - Middle IDACI - Sexual minority - White | 1 | 7 | 21.27 | 11.88 | 33.18 | 21.65 | 13.48 | 31.61 | -0.38 | -6.95 | 5.26 |
| 21221 | Adolescent mental distress at either ages 15 or 17 - Male - Middle IDACI - Sexual minority - White | 1 | 10 | 19.99 | 11.38 | 30.14 | 20.28 | 12.49 | 29.83 | -0.29 | -6.42 | 5.06 |
| 11111 | No adolescent mental distress at either ages 15 or 17 - Male - Lowest IDACI - Heterosexual/straight - White | 9 | 243 | 4.85 | 3.07 | 6.67 | 5.13 | 3.40 | 7.15 | -0.28 | -2.20 | 0.96 |
| 21111 | Adolescent mental distress at either ages 15 or 17 - Male - Lowest IDACI - Heterosexual/straight - White | 12 | 204 | 6.72 | 4.37 | 9.44 | 6.95 | 4.67 | 9.45 | -0.23 | -2.34 | 1.54 |
| 12211 | No adolescent mental distress at either ages 15 or 17 - Female - Middle IDACI - Heterosexual/straight - White | 5 | 110 | 6.33 | 3.80 | 9.21 | 6.55 | 4.24 | 9.37 | -0.22 | -2.49 | 1.45 |
| 11321 | No adolescent mental distress at either ages 15 or 17 - Male - Highest IDACI - Sexual minority - White | 0 | 3 | 20.08 | 10.68 | 35.02 | 20.30 | 11.57 | 33.40 | -0.21 | -6.78 | 5.94 |
| 22124 | Adolescent mental distress at either ages 15 or 17 - Female - Lowest IDACI - Sexual minority - Other (including mixed) | 0 | 2 | 24.20 | 11.36 | 38.94 | 24.40 | 12.85 | 37.92 | -0.21 | -7.38 | 5.81 |
| 22224 | Adolescent mental distress at either ages 15 or 17 - Female - Middle IDACI - Sexual minority - Other (including mixed) | 0 | 2 | 21.66 | 10.52 | 37.31 | 21.86 | 11.08 | 34.99 | -0.20 | -7.51 | 5.76 |
| 22121 | Adolescent mental distress at either ages 15 or 17 - Female - Lowest IDACI - Sexual minority - White | 11 | 39 | 30.22 | 21.25 | 40.59 | 30.40 | 20.78 | 40.02 | -0.18 | -6.29 | 6.77 |
| 21121 | Adolescent mental distress at either ages 15 or 17 - Male - Lowest IDACI - Sexual minority - White | 5 | 25 | 22.49 | 14.41 | 32.27 | 22.67 | 15.03 | 32.04 | -0.17 | -5.28 | 5.04 |
| 22312 | Adolescent mental distress at either ages 15 or 17 - Female - Highest IDACI - Heterosexual/straight - Black | 0 | 47 | 3.84 | 1.17 | 7.83 | 3.98 | 1.27 | 8.13 | -0.14 | -1.51 | 1.10 |
| 12321 | No adolescent mental distress at either ages 15 or 17 - Female - Highest IDACI - Sexual minority - White | 1 | 4 | 27.31 | 15.62 | 43.99 | 27.43 | 16.27 | 41.28 | -0.12 | -6.90 | 7.28 |
| 21322 | Adolescent mental distress at either ages 15 or 17 - Male - Highest IDACI - Sexual minority - Black | 0 | 1 | 9.71 | 2.82 | 20.12 | 9.82 | 3.15 | 20.12 | -0.12 | -3.60 | 3.12 |
| 12314 | No adolescent mental distress at either ages 15 or 17 - Female - Highest IDACI - Heterosexual/straight - Other (including mixed) | 0 | 8 | 6.63 | 2.73 | 12.33 | 6.71 | 3.01 | 11.92 | -0.08 | -2.24 | 2.39 |
| 12313 | No adolescent mental distress at either ages 15 or 17 - Female - Highest IDACI - Heterosexual/straight - Asian | 0 | 51 | 2.92 | 1.38 | 5.02 | 2.99 | 1.49 | 5.07 | -0.07 | -1.13 | 0.88 |
| 21314 | Adolescent mental distress at either ages 15 or 17 - Male - Highest IDACI - Heterosexual/straight - Other (including mixed) | 0 | 12 | 6.17 | 2.53 | 11.42 | 6.24 | 2.80 | 11.20 | -0.07 | -2.08 | 2.06 |
| 22114 | Adolescent mental distress at either ages 15 or 17 - Female - Lowest IDACI - Heterosexual/straight - Other (including mixed) | 1 | 21 | 7.66 | 3.55 | 13.31 | 7.72 | 3.59 | 13.22 | -0.06 | -2.15 | 2.33 |
| 22314 | Adolescent mental distress at either ages 15 or 17 - Female - Highest IDACI - Heterosexual/straight - Other (including mixed) | 1 | 18 | 8.95 | 4.07 | 15.95 | 8.98 | 4.32 | 15.59 | -0.03 | -2.85 | 2.98 |
| 11311 | No adolescent mental distress at either ages 15 or 17 - Male - Highest IDACI - Heterosexual/straight - White | 2 | 33 | 6.06 | 3.31 | 10.57 | 6.09 | 3.54 | 9.94 | -0.03 | -2.00 | 2.17 |
| 22313 | Adolescent mental distress at either ages 15 or 17 - Female - Highest IDACI - Heterosexual/straight - Asian | 3 | 91 | 4.04 | 1.92 | 6.94 | 4.06 | 2.00 | 6.69 | -0.03 | -1.32 | 1.21 |
| 21123 | Adolescent mental distress at either ages 15 or 17 - Male - Lowest IDACI - Sexual minority - Asian | 0 | 1 | 8.69 | 3.70 | 16.53 | 8.72 | 4.09 | 16.36 | -0.02 | -2.79 | 3.04 |
| 21213 | Adolescent mental distress at either ages 15 or 17 - Male - Middle IDACI - Heterosexual/straight - Asian | 0 | 43 | 2.03 | 0.81 | 3.79 | 2.05 | 0.98 | 3.68 | -0.02 | -0.71 | 0.77 |
| 21313 | Adolescent mental distress at either ages 15 or 17 - Male - Highest IDACI - Heterosexual/straight - Asian | 1 | 38 | 2.75 | 1.23 | 4.89 | 2.77 | 1.29 | 4.79 | -0.02 | -0.97 | 0.98 |
| 12212 | No adolescent mental distress at either ages 15 or 17 - Female - Middle IDACI - Heterosexual/straight - Black | 0 | 12 | 2.18 | 0.65 | 4.82 | 2.19 | 0.70 | 4.65 | -0.02 | -0.75 | 0.75 |
| 11112 | No adolescent mental distress at either ages 15 or 17 - Male - Lowest IDACI - Heterosexual/straight - Black | 0 | 3 | 1.72 | 0.42 | 4.21 | 1.72 | 0.51 | 3.96 | 0.00 | -0.61 | 0.57 |
| 11312 | No adolescent mental distress at either ages 15 or 17 - Male - Highest IDACI - Heterosexual/straight - Black | 0 | 23 | 1.99 | 0.58 | 4.23 | 1.99 | 0.61 | 4.08 | 0.01 | -0.72 | 0.81 |
| 22321 | Adolescent mental distress at either ages 15 or 17 - Female - Highest IDACI - Sexual minority - White | 3 | 6 | 34.09 | 19.87 | 49.37 | 34.08 | 21.88 | 47.52 | 0.01 | -8.76 | 7.40 |
| 21113 | Adolescent mental distress at either ages 15 or 17 - Male - Lowest IDACI - Heterosexual/straight - Asian | 0 | 12 | 2.37 | 0.99 | 4.49 | 2.36 | 1.11 | 4.24 | 0.01 | -0.76 | 0.94 |
| 22112 | Adolescent mental distress at either ages 15 or 17 - Female - Lowest IDACI - Heterosexual/straight - Black | 0 | 11 | 3.47 | 0.95 | 7.87 | 3.46 | 0.96 | 7.43 | 0.01 | -0.98 | 1.45 |
| 22214 | Adolescent mental distress at either ages 15 or 17 - Female - Middle IDACI - Heterosexual/straight - Other (including mixed) | 1 | 17 | 6.76 | 3.06 | 12.14 | 6.74 | 3.18 | 11.61 | 0.02 | -2.01 | 1.90 |
| 12111 | No adolescent mental distress at either ages 15 or 17 - Female - Lowest IDACI - Heterosexual/straight - White | 15 | 202 | 7.51 | 5.09 | 10.45 | 7.50 | 5.05 | 10.45 | 0.02 | -1.72 | 2.20 |
| 12113 | No adolescent mental distress at either ages 15 or 17 - Female - Lowest IDACI - Heterosexual/straight - Asian | 0 | 17 | 2.59 | 1.12 | 4.81 | 2.56 | 1.17 | 4.60 | 0.02 | -0.82 | 1.07 |
| 11223 | No adolescent mental distress at either ages 15 or 17 - Male - Middle IDACI - Sexual minority - Asian | 0 | 4 | 5.73 | 2.38 | 11.94 | 5.70 | 2.49 | 11.56 | 0.02 | -2.06 | 2.36 |
| 11121 | No adolescent mental distress at either ages 15 or 17 - Male - Lowest IDACI - Sexual minority - White | 2 | 11 | 17.64 | 10.23 | 27.16 | 17.62 | 10.58 | 26.04 | 0.02 | -5.55 | 5.88 |
| 12224 | No adolescent mental distress at either ages 15 or 17 - Female - Middle IDACI - Sexual minority - Other (including mixed) | 0 | 1 | 17.08 | 7.49 | 30.86 | 17.05 | 7.75 | 29.89 | 0.03 | -4.72 | 5.04 |
| 11313 | No adolescent mental distress at either ages 15 or 17 - Male - Highest IDACI - Heterosexual/straight - Asian | 1 | 50 | 2.05 | 0.91 | 3.87 | 2.03 | 0.96 | 3.52 | 0.03 | -0.62 | 0.79 |
| 22211 | Adolescent mental distress at either ages 15 or 17 - Female - Middle IDACI - Heterosexual/straight - White | 17 | 196 | 8.83 | 6.00 | 11.96 | 8.80 | 5.86 | 12.21 | 0.03 | -2.31 | 2.60 |
| 12312 | No adolescent mental distress at either ages 15 or 17 - Female - Highest IDACI - Heterosexual/straight - Black | 1 | 26 | 2.96 | 0.89 | 6.15 | 2.93 | 0.94 | 6.02 | 0.03 | -1.02 | 1.19 |
| 21212 | Adolescent mental distress at either ages 15 or 17 - Male - Middle IDACI - Heterosexual/straight - Black | 0 | 10 | 2.05 | 0.60 | 4.51 | 2.02 | 0.60 | 4.47 | 0.03 | -0.65 | 0.83 |
| 12323 | No adolescent mental distress at either ages 15 or 17 - Female - Highest IDACI - Sexual minority - Asian | 0 | 2 | 10.92 | 4.51 | 20.40 | 10.89 | 4.97 | 19.24 | 0.03 | -3.53 | 3.94 |
| 22113 | Adolescent mental distress at either ages 15 or 17 - Female - Lowest IDACI - Heterosexual/straight - Asian | 1 | 37 | 3.52 | 1.57 | 6.32 | 3.48 | 1.70 | 5.95 | 0.03 | -0.91 | 1.33 |
| 21112 | Adolescent mental distress at either ages 15 or 17 - Male - Lowest IDACI - Heterosexual/straight - Black | 0 | 4 | 2.38 | 0.62 | 5.25 | 2.35 | 0.65 | 5.13 | 0.04 | -0.77 | 0.94 |
| 12222 | No adolescent mental distress at either ages 15 or 17 - Female - Middle IDACI - Sexual minority - Black | 0 | 1 | 8.14 | 2.02 | 17.96 | 8.10 | 2.42 | 17.45 | 0.04 | -2.48 | 3.25 |
| 11211 | No adolescent mental distress at either ages 15 or 17 - Male - Middle IDACI - Heterosexual/straight - White | 7 | 139 | 4.52 | 2.81 | 6.98 | 4.48 | 2.88 | 6.39 | 0.05 | -1.43 | 1.52 |
| 22222 | Adolescent mental distress at either ages 15 or 17 - Female - Middle IDACI - Sexual minority - Black | 0 | 2 | 10.74 | 3.04 | 22.96 | 10.69 | 3.44 | 22.13 | 0.05 | -2.96 | 3.74 |
| 21214 | Adolescent mental distress at either ages 15 or 17 - Male - Middle IDACI - Heterosexual/straight - Other (including mixed) | 0 | 8 | 4.69 | 2.00 | 8.97 | 4.64 | 2.06 | 8.63 | 0.05 | -1.33 | 1.79 |
| 11113 | No adolescent mental distress at either ages 15 or 17 - Male - Lowest IDACI - Heterosexual/straight - Asian | 1 | 33 | 1.79 | 0.75 | 3.51 | 1.73 | 0.80 | 3.14 | 0.06 | -0.51 | 1.07 |
| 11213 | No adolescent mental distress at either ages 15 or 17 - Male - Middle IDACI - Heterosexual/straight - Asian | 2 | 60 | 1.56 | 0.66 | 2.93 | 1.49 | 0.67 | 2.79 | 0.06 | -0.36 | 0.80 |
| 11212 | No adolescent mental distress at either ages 15 or 17 - Male - Middle IDACI - Heterosexual/straight - Black | 0 | 7 | 1.55 | 0.42 | 3.72 | 1.48 | 0.44 | 3.22 | 0.07 | -0.55 | 0.85 |
| 21222 | Adolescent mental distress at either ages 15 or 17 - Male - Middle IDACI - Sexual minority - Black | 0 | 1 | 7.55 | 2.13 | 17.75 | 7.48 | 2.18 | 16.42 | 0.07 | -2.27 | 3.42 |
| 21223 | Adolescent mental distress at either ages 15 or 17 - Male - Middle IDACI - Sexual minority - Asian | 0 | 2 | 7.72 | 2.96 | 14.68 | 7.64 | 3.28 | 14.04 | 0.08 | -2.35 | 3.01 |
| 21323 | Adolescent mental distress at either ages 15 or 17 - Male - Highest IDACI - Sexual minority - Asian | 0 | 3 | 10.18 | 4.51 | 19.07 | 10.10 | 4.56 | 17.85 | 0.08 | -2.66 | 4.21 |
| 22213 | Adolescent mental distress at either ages 15 or 17 - Female - Middle IDACI - Heterosexual/straight - Asian | 3 | 69 | 3.11 | 1.44 | 5.49 | 3.02 | 1.41 | 5.16 | 0.09 | -0.81 | 1.46 |
| 12114 | No adolescent mental distress at either ages 15 or 17 - Female - Lowest IDACI - Heterosexual/straight - Other (including mixed) | 1 | 12 | 5.84 | 2.40 | 11.04 | 5.76 | 2.63 | 10.26 | 0.09 | -1.73 | 2.55 |
| 21312 | Adolescent mental distress at either ages 15 or 17 - Male - Highest IDACI - Heterosexual/straight - Black | 1 | 11 | 2.81 | 0.80 | 6.37 | 2.71 | 0.87 | 5.78 | 0.10 | -0.74 | 1.28 |
| 21114 | Adolescent mental distress at either ages 15 or 17 - Male - Lowest IDACI - Heterosexual/straight - Other (including mixed) | 1 | 18 | 5.43 | 2.38 | 9.86 | 5.32 | 2.50 | 9.42 | 0.11 | -1.34 | 2.10 |
| 22324 | Adolescent mental distress at either ages 15 or 17 - Female - Highest IDACI - Sexual minority - Other (including mixed) | 1 | 3 | 27.64 | 14.35 | 45.07 | 27.52 | 14.67 | 42.60 | 0.11 | -6.40 | 7.91 |
| 12213 | No adolescent mental distress at either ages 15 or 17 - Female - Middle IDACI - Heterosexual/straight - Asian | 2 | 34 | 2.33 | 1.08 | 4.41 | 2.22 | 1.04 | 4.01 | 0.12 | -0.53 | 1.33 |
| 11214 | No adolescent mental distress at either ages 15 or 17 - Male - Middle IDACI - Heterosexual/straight - Other (including mixed) | 1 | 11 | 3.53 | 1.37 | 7.33 | 3.41 | 1.51 | 6.40 | 0.12 | -1.10 | 1.97 |
| 11314 | No adolescent mental distress at either ages 15 or 17 - Male - Highest IDACI - Heterosexual/straight - Other (including mixed) | 1 | 7 | 4.73 | 1.83 | 9.37 | 4.61 | 2.00 | 8.60 | 0.12 | -1.50 | 2.44 |
| 22212 | Adolescent mental distress at either ages 15 or 17 - Female - Middle IDACI - Heterosexual/straight - Black | 2 | 23 | 3.13 | 0.98 | 6.87 | 2.98 | 0.94 | 6.31 | 0.14 | -0.77 | 1.62 |
| 11114 | No adolescent mental distress at either ages 15 or 17 - Male - Lowest IDACI - Heterosexual/straight - Other (including mixed) | 1 | 9 | 4.08 | 1.77 | 7.88 | 3.93 | 1.74 | 7.03 | 0.15 | -0.99 | 2.19 |
| 11224 | No adolescent mental distress at either ages 15 or 17 - Male - Middle IDACI - Sexual minority - Other (including mixed) | 0 | 1 | 12.38 | 5.16 | 25.69 | 12.17 | 5.29 | 22.76 | 0.21 | -3.46 | 5.47 |
| 12311 | No adolescent mental distress at either ages 15 or 17 - Female - Highest IDACI - Heterosexual/straight - White | 4 | 33 | 9.05 | 5.30 | 14.58 | 8.81 | 5.37 | 13.61 | 0.24 | -2.53 | 3.35 |
| 12214 | No adolescent mental distress at either ages 15 or 17 - Female - Middle IDACI - Heterosexual/straight - Other (including mixed) | 1 | 9 | 5.26 | 2.20 | 10.45 | 5.01 | 2.29 | 9.17 | 0.26 | -1.37 | 3.34 |
| 21224 | Adolescent mental distress at either ages 15 or 17 - Male - Middle IDACI - Sexual minority - Other (including mixed) | 0 | 1 | 16.15 | 6.94 | 29.33 | 15.89 | 7.24 | 27.73 | 0.26 | -3.94 | 5.34 |
| 21311 | Adolescent mental distress at either ages 15 or 17 - Male - Highest IDACI - Heterosexual/straight - White | 3 | 22 | 8.50 | 4.61 | 14.21 | 8.21 | 4.72 | 12.79 | 0.28 | -2.31 | 3.58 |
| 12324 | No adolescent mental distress at either ages 15 or 17 - Female - Highest IDACI - Sexual minority - Other (including mixed) | 1 | 1 | 22.16 | 10.28 | 38.42 | 21.82 | 10.67 | 36.19 | 0.34 | -5.31 | 7.75 |
| 21211 | Adolescent mental distress at either ages 15 or 17 - Male - Middle IDACI - Heterosexual/straight - White | 8 | 86 | 6.47 | 3.95 | 9.75 | 6.08 | 3.88 | 8.93 | 0.38 | -1.26 | 3.26 |
| 22323 | Adolescent mental distress at either ages 15 or 17 - Female - Highest IDACI - Sexual minority - Asian | 1 | 2 | 14.69 | 6.56 | 27.60 | 14.27 | 7.00 | 23.76 | 0.42 | -3.62 | 7.81 |
| 12121 | No adolescent mental distress at either ages 15 or 17 - Female - Lowest IDACI - Sexual minority - White | 2 | 5 | 24.62 | 14.97 | 37.13 | 24.18 | 15.16 | 34.43 | 0.44 | -5.23 | 8.10 |
| 11323 | No adolescent mental distress at either ages 15 or 17 - Male - Highest IDACI - Sexual minority - Asian | 1 | 2 | 8.11 | 3.38 | 16.92 | 7.60 | 3.37 | 14.18 | 0.51 | -1.96 | 5.13 |
| 11221 | No adolescent mental distress at either ages 15 or 17 - Male - Middle IDACI - Sexual minority - White | 2 | 6 | 16.21 | 8.84 | 27.26 | 15.65 | 9.15 | 24.20 | 0.56 | -3.55 | 6.81 |
| 22322 | Adolescent mental distress at either ages 15 or 17 - Female - Highest IDACI - Sexual minority - Black | 2 | 2 | 14.59 | 4.80 | 29.64 | 13.86 | 4.75 | 27.03 | 0.73 | -2.97 | 7.34 |
| 21321 | Adolescent mental distress at either ages 15 or 17 - Male - Highest IDACI - Sexual minority - White | 2 | 5 | 26.61 | 15.01 | 43.69 | 25.84 | 15.34 | 39.75 | 0.77 | -4.37 | 10.06 |
| 21324 | Adolescent mental distress at either ages 15 or 17 - Male - Highest IDACI - Sexual minority - Other (including mixed) | 1 | 1 | 21.23 | 9.26 | 39.15 | 20.44 | 10.02 | 34.33 | 0.79 | -4.63 | 9.85 |
| 22111 | Adolescent mental distress at either ages 15 or 17 - Female - Lowest IDACI - Heterosexual/straight - White | 51 | 391 | 11.24 | 8.68 | 14.31 | 10.03 | 6.69 | 12.91 | 1.21 | -0.94 | 5.87 |

*Note.* I+M effects = interaction effects and main effects conflated; M effects = main effects only; I effects = differences between intersections and main effects.

## Table A5. Predicted incidence (%) of having self-harmed in the last year at age 25 for individuals who did not attend university by social strata based on conflated interaction effects and main effects, main effects only, and differences between intersections and main effects, ranked according to the extent to which each interaction effect differs from what is explained by the main effects alone (i.e. the residuals)

| Stratum | Stratum Label | Number of cases | Number of individuals | Predicted incidence (I+M effects) | Low 95% CI predicted incidence (I+M effects) | High 95% CI predicted incidence (I+M effects) | Predicted incidence (M effects) | Low 95% CI predicted incidence (M effects) | High 95% CI predicted incidence (M effects) | Predicted incidence (I effects) | Low 95% CI predicted incidence (I effects) | High 95% CI predicted incidence (I effects) |
| --- | --- | --- | --- | --- | --- | --- | --- | --- | --- | --- | --- | --- |
| 22121 | Adolescent mental distress at either ages 15 or 17 - Female - Lowest IDACI - Sexual minority - White | 2 | 17 | 24.78 | 12.91 | 36.97 | 26.21 | 15.46 | 38.87 | -1.43 | -12.87 | 4.96 |
| 21321 | Adolescent mental distress at either ages 15 or 17 - Male - Highest IDACI - Sexual minority - White | 0 | 8 | 21.31 | 10.28 | 34.80 | 22.29 | 12.01 | 35.18 | -0.99 | -9.76 | 4.54 |
| 21221 | Adolescent mental distress at either ages 15 or 17 - Male - Middle IDACI - Sexual minority - White | 2 | 13 | 23.80 | 12.54 | 37.20 | 24.34 | 14.73 | 36.23 | -0.54 | -8.50 | 6.59 |
| 22211 | Adolescent mental distress at either ages 15 or 17 - Female - Middle IDACI - Heterosexual/straight - White | 11 | 231 | 5.80 | 3.43 | 8.51 | 6.16 | 3.79 | 9.26 | -0.36 | -3.24 | 1.57 |
| 22311 | Adolescent mental distress at either ages 15 or 17 - Female - Highest IDACI - Heterosexual/straight - White | 5 | 133 | 5.18 | 2.76 | 8.29 | 5.52 | 3.32 | 8.70 | -0.34 | -3.00 | 1.31 |
| 22322 | Adolescent mental distress at either ages 15 or 17 - Female - Highest IDACI - Sexual minority - Black | 0 | 2 | 25.73 | 7.89 | 50.92 | 25.91 | 8.46 | 50.05 | -0.18 | -7.98 | 8.67 |
| 12221 | No adolescent mental distress at either ages 15 or 17 - Female - Middle IDACI - Sexual minority - White | 1 | 12 | 12.10 | 5.22 | 21.70 | 12.25 | 5.99 | 20.63 | -0.15 | -5.21 | 3.91 |
| 22214 | Adolescent mental distress at either ages 15 or 17 - Female - Middle IDACI - Heterosexual/straight - Other (including mixed) | 0 | 13 | 5.92 | 1.59 | 12.77 | 6.07 | 1.83 | 12.30 | -0.15 | -3.06 | 2.29 |
| 21121 | Adolescent mental distress at either ages 15 or 17 - Male - Lowest IDACI - Sexual minority - White | 2 | 12 | 20.12 | 10.23 | 32.23 | 20.24 | 11.73 | 30.80 | -0.13 | -7.26 | 6.85 |
| 12111 | No adolescent mental distress at either ages 15 or 17 - Female - Lowest IDACI - Heterosexual/straight - White | 0 | 150 | 1.45 | 0.56 | 2.61 | 1.56 | 0.78 | 2.73 | -0.11 | -0.96 | 0.41 |
| 22114 | Adolescent mental distress at either ages 15 or 17 - Female - Lowest IDACI - Heterosexual/straight - Other (including mixed) | 0 | 15 | 4.78 | 1.32 | 11.30 | 4.85 | 1.49 | 10.73 | -0.08 | -2.31 | 1.99 |
| 11111 | No adolescent mental distress at either ages 15 or 17 - Male - Lowest IDACI - Heterosexual/straight - White | 1 | 225 | 1.06 | 0.39 | 1.91 | 1.12 | 0.54 | 2.00 | -0.06 | -0.53 | 0.29 |
| 21312 | Adolescent mental distress at either ages 15 or 17 - Male - Highest IDACI - Heterosexual/straight - Black | 0 | 14 | 3.66 | 0.78 | 9.18 | 3.71 | 0.87 | 8.92 | -0.06 | -1.80 | 1.60 |
| 22312 | Adolescent mental distress at either ages 15 or 17 - Female - Highest IDACI - Heterosexual/straight - Black | 1 | 25 | 5.03 | 1.31 | 11.42 | 5.07 | 1.37 | 11.81 | -0.05 | -2.55 | 2.33 |
| 21111 | Adolescent mental distress at either ages 15 or 17 - Male - Lowest IDACI - Heterosexual/straight - White | 5 | 147 | 3.51 | 1.90 | 5.57 | 3.55 | 2.03 | 5.68 | -0.04 | -1.39 | 1.10 |
| 21114 | Adolescent mental distress at either ages 15 or 17 - Male - Lowest IDACI - Heterosexual/straight - Other (including mixed) | 0 | 4 | 3.53 | 0.89 | 8.62 | 3.55 | 0.97 | 8.29 | -0.02 | -1.48 | 1.53 |
| 12213 | No adolescent mental distress at either ages 15 or 17 - Female - Middle IDACI - Heterosexual/straight - Asian | 0 | 18 | 1.11 | 0.31 | 2.91 | 1.12 | 0.31 | 2.78 | -0.01 | -0.47 | 0.53 |
| 11222 | No adolescent mental distress at either ages 15 or 17 - Male - Middle IDACI - Sexual minority - Black | 0 | 1 | 8.45 | 1.76 | 20.92 | 8.46 | 1.87 | 20.46 | -0.01 | -3.54 | 3.74 |
| 11214 | No adolescent mental distress at either ages 15 or 17 - Male - Middle IDACI - Heterosexual/straight - Other (including mixed) | 0 | 10 | 1.42 | 0.32 | 3.56 | 1.42 | 0.35 | 3.36 | -0.01 | -0.64 | 0.61 |
| 12314 | No adolescent mental distress at either ages 15 or 17 - Female - Highest IDACI - Heterosexual/straight - Other (including mixed) | 0 | 11 | 1.75 | 0.40 | 4.30 | 1.75 | 0.48 | 4.25 | 0.00 | -0.77 | 0.89 |
| 21213 | Adolescent mental distress at either ages 15 or 17 - Male - Middle IDACI - Heterosexual/straight - Asian | 0 | 17 | 2.56 | 0.70 | 6.01 | 2.56 | 0.81 | 5.82 | 0.00 | -1.17 | 1.39 |
| 21214 | Adolescent mental distress at either ages 15 or 17 - Male - Middle IDACI - Heterosexual/straight - Other (including mixed) | 0 | 7 | 4.45 | 1.09 | 9.84 | 4.45 | 1.16 | 9.70 | 0.00 | -2.07 | 2.44 |
| 12323 | No adolescent mental distress at either ages 15 or 17 - Female - Highest IDACI - Sexual minority - Asian | 0 | 3 | 6.52 | 1.59 | 16.41 | 6.52 | 1.74 | 16.08 | 0.00 | -2.63 | 3.04 |
| 21113 | Adolescent mental distress at either ages 15 or 17 - Male - Lowest IDACI - Heterosexual/straight - Asian | 0 | 11 | 2.04 | 0.52 | 4.78 | 2.04 | 0.59 | 4.51 | 0.01 | -0.90 | 1.05 |
| 21314 | Adolescent mental distress at either ages 15 or 17 - Male - Highest IDACI - Heterosexual/straight - Other (including mixed) | 0 | 7 | 3.96 | 1.02 | 9.39 | 3.96 | 1.14 | 8.71 | 0.01 | -1.88 | 1.75 |
| 11313 | No adolescent mental distress at either ages 15 or 17 - Male - Highest IDACI - Heterosexual/straight - Asian | 0 | 39 | 0.71 | 0.17 | 1.72 | 0.70 | 0.20 | 1.64 | 0.01 | -0.31 | 0.35 |
| 21212 | Adolescent mental distress at either ages 15 or 17 - Male - Middle IDACI - Heterosexual/straight - Black | 0 | 4 | 4.23 | 0.97 | 11.19 | 4.22 | 1.06 | 10.18 | 0.01 | -1.75 | 2.27 |
| 11314 | No adolescent mental distress at either ages 15 or 17 - Male - Highest IDACI - Heterosexual/straight - Other (including mixed) | 0 | 12 | 1.28 | 0.26 | 3.30 | 1.27 | 0.30 | 3.08 | 0.01 | -0.48 | 0.66 |
| 11212 | No adolescent mental distress at either ages 15 or 17 - Male - Middle IDACI - Heterosexual/straight - Black | 0 | 9 | 1.36 | 0.27 | 3.76 | 1.34 | 0.29 | 3.61 | 0.01 | -0.64 | 0.83 |
| 11213 | No adolescent mental distress at either ages 15 or 17 - Male - Middle IDACI - Heterosexual/straight - Asian | 0 | 16 | 0.82 | 0.20 | 2.13 | 0.80 | 0.21 | 2.01 | 0.01 | -0.37 | 0.49 |
| 12113 | No adolescent mental distress at either ages 15 or 17 - Female - Lowest IDACI - Heterosexual/straight - Asian | 0 | 3 | 0.91 | 0.22 | 2.32 | 0.89 | 0.23 | 2.17 | 0.02 | -0.36 | 0.53 |
| 11113 | No adolescent mental distress at either ages 15 or 17 - Male - Lowest IDACI - Heterosexual/straight - Asian | 0 | 9 | 0.65 | 0.14 | 1.70 | 0.64 | 0.16 | 1.53 | 0.02 | -0.23 | 0.38 |
| 12114 | No adolescent mental distress at either ages 15 or 17 - Female - Lowest IDACI - Heterosexual/straight - Other (including mixed) | 0 | 1 | 1.58 | 0.38 | 4.01 | 1.56 | 0.42 | 3.74 | 0.02 | -0.67 | 1.08 |
| 11312 | No adolescent mental distress at either ages 15 or 17 - Male - Highest IDACI - Heterosexual/straight - Black | 0 | 13 | 1.20 | 0.23 | 3.55 | 1.18 | 0.25 | 3.16 | 0.02 | -0.55 | 0.89 |
| 21123 | Adolescent mental distress at either ages 15 or 17 - Male - Lowest IDACI - Sexual minority - Asian | 0 | 1 | 12.51 | 3.49 | 28.21 | 12.49 | 3.55 | 27.10 | 0.02 | -4.77 | 5.78 |
| 12312 | No adolescent mental distress at either ages 15 or 17 - Female - Highest IDACI - Heterosexual/straight - Black | 0 | 12 | 1.66 | 0.34 | 4.59 | 1.64 | 0.35 | 4.24 | 0.02 | -0.67 | 0.92 |
| 11114 | No adolescent mental distress at either ages 15 or 17 - Male - Lowest IDACI - Heterosexual/straight - Other (including mixed) | 0 | 7 | 1.16 | 0.24 | 3.11 | 1.13 | 0.28 | 2.92 | 0.03 | -0.49 | 0.79 |
| 12212 | No adolescent mental distress at either ages 15 or 17 - Female - Middle IDACI - Heterosexual/straight - Black | 0 | 3 | 1.89 | 0.38 | 5.29 | 1.86 | 0.42 | 4.70 | 0.03 | -0.84 | 1.20 |
| 11112 | No adolescent mental distress at either ages 15 or 17 - Male - Lowest IDACI - Heterosexual/straight - Black | 0 | 2 | 1.11 | 0.19 | 3.15 | 1.08 | 0.21 | 3.04 | 0.03 | -0.45 | 0.68 |
| 12214 | No adolescent mental distress at either ages 15 or 17 - Female - Middle IDACI - Heterosexual/straight - Other (including mixed) | 0 | 5 | 2.01 | 0.46 | 4.71 | 1.97 | 0.50 | 4.55 | 0.04 | -0.86 | 0.96 |
| 21313 | Adolescent mental distress at either ages 15 or 17 - Male - Highest IDACI - Heterosexual/straight - Asian | 1 | 31 | 2.28 | 0.66 | 5.25 | 2.24 | 0.73 | 4.83 | 0.04 | -0.86 | 1.25 |
| 12313 | No adolescent mental distress at either ages 15 or 17 - Female - Highest IDACI - Heterosexual/straight - Asian | 1 | 39 | 1.02 | 0.28 | 2.48 | 0.98 | 0.30 | 2.30 | 0.04 | -0.35 | 0.60 |
| 12211 | No adolescent mental distress at either ages 15 or 17 - Female - Middle IDACI - Heterosexual/straight - White | 3 | 131 | 2.02 | 0.99 | 3.72 | 1.98 | 1.07 | 3.49 | 0.04 | -0.74 | 1.08 |
| 22212 | Adolescent mental distress at either ages 15 or 17 - Female - Middle IDACI - Heterosexual/straight - Black | 0 | 5 | 5.79 | 1.45 | 14.31 | 5.75 | 1.55 | 13.70 | 0.04 | -2.63 | 2.83 |
| 11311 | No adolescent mental distress at either ages 15 or 17 - Male - Highest IDACI - Heterosexual/straight - White | 2 | 110 | 1.32 | 0.56 | 2.57 | 1.27 | 0.59 | 2.41 | 0.05 | -0.45 | 0.80 |
| 22213 | Adolescent mental distress at either ages 15 or 17 - Female - Middle IDACI - Heterosexual/straight - Asian | 1 | 20 | 3.59 | 1.12 | 7.88 | 3.54 | 1.16 | 7.81 | 0.05 | -1.43 | 1.88 |
| 22113 | Adolescent mental distress at either ages 15 or 17 - Female - Lowest IDACI - Heterosexual/straight - Asian | 0 | 6 | 2.88 | 0.79 | 6.60 | 2.82 | 0.88 | 6.16 | 0.06 | -1.13 | 1.71 |
| 11323 | No adolescent mental distress at either ages 15 or 17 - Male - Highest IDACI - Sexual minority - Asian | 0 | 1 | 4.82 | 1.12 | 12.00 | 4.75 | 1.18 | 11.55 | 0.07 | -1.63 | 2.24 |
| 12222 | No adolescent mental distress at either ages 15 or 17 - Female - Middle IDACI - Sexual minority - Black | 0 | 1 | 11.42 | 2.49 | 28.56 | 11.34 | 2.60 | 26.13 | 0.08 | -5.48 | 5.77 |
| 12311 | No adolescent mental distress at either ages 15 or 17 - Female - Highest IDACI - Heterosexual/straight - White | 2 | 80 | 1.86 | 0.83 | 3.57 | 1.77 | 0.87 | 3.26 | 0.09 | -0.60 | 1.20 |
| 11211 | No adolescent mental distress at either ages 15 or 17 - Male - Middle IDACI - Heterosexual/straight - White | 4 | 195 | 1.50 | 0.71 | 2.66 | 1.41 | 0.72 | 2.52 | 0.09 | -0.41 | 0.91 |
| 11321 | No adolescent mental distress at either ages 15 or 17 - Male - Highest IDACI - Sexual minority - White | 0 | 3 | 8.28 | 3.14 | 16.29 | 8.19 | 3.60 | 15.24 | 0.09 | -2.67 | 3.42 |
| 22321 | Adolescent mental distress at either ages 15 or 17 - Female - Highest IDACI - Sexual minority - White | 3 | 11 | 28.69 | 16.03 | 44.01 | 28.60 | 17.33 | 42.49 | 0.09 | -8.50 | 11.53 |
| 11223 | No adolescent mental distress at either ages 15 or 17 - Male - Middle IDACI - Sexual minority - Asian | 0 | 1 | 5.46 | 1.32 | 13.21 | 5.35 | 1.28 | 13.06 | 0.11 | -2.07 | 2.77 |
| 22324 | Adolescent mental distress at either ages 15 or 17 - Female - Highest IDACI - Sexual minority - Other (including mixed) | 0 | 1 | 27.57 | 8.99 | 50.41 | 27.47 | 10.30 | 50.30 | 0.11 | -8.46 | 9.97 |
| 22313 | Adolescent mental distress at either ages 15 or 17 - Female - Highest IDACI - Heterosexual/straight - Asian | 3 | 70 | 3.23 | 1.12 | 6.62 | 3.10 | 1.05 | 6.36 | 0.12 | -1.25 | 1.97 |
| 22112 | Adolescent mental distress at either ages 15 or 17 - Female - Lowest IDACI - Heterosexual/straight - Black | 1 | 4 | 4.85 | 1.03 | 12.98 | 4.64 | 1.03 | 11.59 | 0.20 | -1.59 | 3.21 |
| 21112 | Adolescent mental distress at either ages 15 or 17 - Male - Lowest IDACI - Heterosexual/straight - Black | 1 | 1 | 3.62 | 0.70 | 9.87 | 3.39 | 0.69 | 8.79 | 0.23 | -0.94 | 2.74 |
| 22111 | Adolescent mental distress at either ages 15 or 17 - Female - Lowest IDACI - Heterosexual/straight - White | 15 | 238 | 5.16 | 3.26 | 7.79 | 4.91 | 3.09 | 7.35 | 0.26 | -1.46 | 2.64 |
| 21211 | Adolescent mental distress at either ages 15 or 17 - Male - Middle IDACI - Heterosexual/straight - White | 8 | 128 | 4.77 | 2.69 | 7.74 | 4.48 | 2.51 | 7.16 | 0.30 | -1.23 | 2.68 |
| 21311 | Adolescent mental distress at either ages 15 or 17 - Male - Highest IDACI - Heterosexual/straight - White | 5 | 72 | 4.36 | 2.26 | 7.72 | 4.01 | 2.16 | 6.62 | 0.35 | -1.11 | 3.22 |
| 11221 | No adolescent mental distress at either ages 15 or 17 - Male - Middle IDACI - Sexual minority - White | 2 | 12 | 9.43 | 4.29 | 17.35 | 9.05 | 4.23 | 16.15 | 0.38 | -2.85 | 5.34 |
| 11121 | No adolescent mental distress at either ages 15 or 17 - Male - Lowest IDACI - Sexual minority - White | 2 | 12 | 7.67 | 3.34 | 14.51 | 7.28 | 3.32 | 12.98 | 0.39 | -1.92 | 4.82 |
| 22314 | Adolescent mental distress at either ages 15 or 17 - Female - Highest IDACI - Heterosexual/straight - Other (including mixed) | 3 | 22 | 5.87 | 1.69 | 12.53 | 5.40 | 1.70 | 11.52 | 0.47 | -1.41 | 4.20 |
| 12321 | No adolescent mental distress at either ages 15 or 17 - Female - Highest IDACI - Sexual minority - White | 1 | 3 | 11.63 | 4.66 | 23.66 | 11.11 | 5.16 | 20.38 | 0.52 | -3.40 | 7.56 |
| 12121 | No adolescent mental distress at either ages 15 or 17 - Female - Lowest IDACI - Sexual minority - White | 2 | 9 | 10.58 | 4.86 | 19.83 | 9.93 | 4.70 | 17.55 | 0.65 | -2.37 | 7.09 |
| 22222 | Adolescent mental distress at either ages 15 or 17 - Female - Middle IDACI - Sexual minority - Black | 1 | 1 | 29.12 | 8.45 | 56.60 | 28.27 | 8.79 | 51.93 | 0.84 | -7.70 | 11.57 |
| 22124 | Adolescent mental distress at either ages 15 or 17 - Female - Lowest IDACI - Sexual minority - Other (including mixed) | 2 | 2 | 26.39 | 8.03 | 51.82 | 25.31 | 8.49 | 47.33 | 1.08 | -6.23 | 13.72 |
| 22221 | Adolescent mental distress at either ages 15 or 17 - Female - Middle IDACI - Sexual minority - White | 8 | 19 | 32.57 | 20.51 | 46.78 | 31.04 | 20.03 | 44.51 | 1.53 | -6.37 | 13.99 |

*Note.* I+M effects = interaction effects and main effects conflated; M effects = main effects only; I effects = differences between intersections and main effects.

## Table A6. Predicted incidence (%) of having self-harmed in the last year at age 25 for individuals who attended university by social strata based on conflated interaction effects and main effects, main effects only, and differences between intersections and main effects, ranked according to the extent to which each interaction effect differs from what is explained by the main effects alone (i.e. the residuals)

| Stratum | Stratum Label | Number of cases | Number of individuals | Predicted incidence (I+M effects) | Low 95% CI predicted incidence (I+M effects) | High 95% CI predicted incidence (I+M effects) | Predicted incidence (M effects) | Low 95% CI predicted incidence (M effects) | High 95% CI predicted incidence (M effects) | Predicted incidence (I effects) | Low 95% CI predicted incidence (I effects) | High 95% CI predicted incidence (I effects) |
| --- | --- | --- | --- | --- | --- | --- | --- | --- | --- | --- | --- | --- |
| 21121 | Adolescent mental distress at either ages 15 or 17 - Male - Lowest IDACI - Sexual minority - White | 2 | 25 | 12.33 | 6.22 | 20.46 | 12.90 | 7.02 | 20.75 | -0.57 | -6.73 | 3.91 |
| 22221 | Adolescent mental distress at either ages 15 or 17 - Female - Middle IDACI - Sexual minority - White | 1 | 15 | 12.92 | 5.91 | 21.56 | 13.45 | 6.92 | 22.89 | -0.53 | -7.43 | 4.14 |
| 22314 | Adolescent mental distress at either ages 15 or 17 - Female - Highest IDACI - Heterosexual/straight - Other (including mixed) | 0 | 18 | 8.06 | 2.47 | 15.68 | 8.58 | 3.40 | 16.34 | -0.53 | -5.69 | 2.02 |
| 22121 | Adolescent mental distress at either ages 15 or 17 - Female - Lowest IDACI - Sexual minority - White | 5 | 38 | 15.37 | 8.18 | 24.19 | 15.88 | 8.94 | 24.64 | -0.51 | -8.64 | 4.37 |
| 21114 | Adolescent mental distress at either ages 15 or 17 - Male - Lowest IDACI - Heterosexual/straight - Other (including mixed) | 0 | 18 | 6.40 | 2.11 | 12.41 | 6.69 | 2.83 | 12.72 | -0.28 | -3.78 | 2.00 |
| 22124 | Adolescent mental distress at either ages 15 or 17 - Female - Lowest IDACI - Sexual minority - Other (including mixed) | 0 | 2 | 24.62 | 9.84 | 45.42 | 24.89 | 11.17 | 43.13 | -0.27 | -11.92 | 10.09 |
| 22213 | Adolescent mental distress at either ages 15 or 17 - Female - Middle IDACI - Heterosexual/straight - Asian | 0 | 68 | 2.57 | 0.79 | 5.03 | 2.75 | 1.04 | 5.32 | -0.18 | -1.84 | 0.78 |
| 21323 | Adolescent mental distress at either ages 15 or 17 - Male - Highest IDACI - Sexual minority - Asian | 0 | 3 | 9.22 | 2.84 | 18.94 | 9.29 | 3.55 | 18.14 | -0.07 | -4.43 | 4.13 |
| 21213 | Adolescent mental distress at either ages 15 or 17 - Male - Middle IDACI - Heterosexual/straight - Asian | 0 | 44 | 2.11 | 0.56 | 4.50 | 2.18 | 0.79 | 4.41 | -0.07 | -1.23 | 0.78 |
| 21311 | Adolescent mental distress at either ages 15 or 17 - Male - Highest IDACI - Heterosexual/straight - White | 0 | 21 | 4.02 | 1.46 | 7.82 | 4.09 | 1.67 | 8.02 | -0.07 | -2.11 | 1.95 |
| 12221 | No adolescent mental distress at either ages 15 or 17 - Female - Middle IDACI - Sexual minority - White | 0 | 7 | 5.49 | 1.87 | 11.14 | 5.56 | 2.24 | 10.83 | -0.07 | -2.60 | 2.69 |
| 21313 | Adolescent mental distress at either ages 15 or 17 - Male - Highest IDACI - Heterosexual/straight - Asian | 0 | 37 | 2.64 | 0.83 | 5.61 | 2.71 | 1.03 | 5.42 | -0.07 | -1.50 | 1.18 |
| 12211 | No adolescent mental distress at either ages 15 or 17 - Female - Middle IDACI - Heterosexual/straight - White | 0 | 110 | 1.49 | 0.46 | 2.80 | 1.54 | 0.68 | 2.77 | -0.06 | -0.76 | 0.57 |
| 12311 | No adolescent mental distress at either ages 15 or 17 - Female - Highest IDACI - Heterosexual/straight - White | 0 | 33 | 1.93 | 0.56 | 3.96 | 1.98 | 0.76 | 4.00 | -0.05 | -0.90 | 0.88 |
| 21111 | Adolescent mental distress at either ages 15 or 17 - Male - Lowest IDACI - Heterosexual/straight - White | 7 | 202 | 3.80 | 2.12 | 6.07 | 3.85 | 2.18 | 6.20 | -0.05 | -1.58 | 1.46 |
| 21211 | Adolescent mental distress at either ages 15 or 17 - Male - Middle IDACI - Heterosexual/straight - White | 2 | 85 | 3.16 | 1.41 | 5.70 | 3.20 | 1.65 | 5.62 | -0.04 | -1.66 | 1.63 |
| 22323 | Adolescent mental distress at either ages 15 or 17 - Female - Highest IDACI - Sexual minority - Asian | 0 | 2 | 11.44 | 3.61 | 22.75 | 11.48 | 4.80 | 21.38 | -0.04 | -5.45 | 6.35 |
| 12111 | No adolescent mental distress at either ages 15 or 17 - Female - Lowest IDACI - Heterosexual/straight - White | 3 | 199 | 1.84 | 0.80 | 3.12 | 1.87 | 0.87 | 3.21 | -0.03 | -0.90 | 0.77 |
| 11313 | No adolescent mental distress at either ages 15 or 17 - Male - Highest IDACI - Heterosexual/straight - Asian | 0 | 49 | 1.00 | 0.28 | 2.14 | 1.02 | 0.35 | 2.08 | -0.02 | -0.48 | 0.42 |
| 11311 | No adolescent mental distress at either ages 15 or 17 - Male - Highest IDACI - Heterosexual/straight - White | 0 | 32 | 1.54 | 0.46 | 3.31 | 1.55 | 0.58 | 3.11 | -0.01 | -0.72 | 0.86 |
| 12114 | No adolescent mental distress at either ages 15 or 17 - Female - Lowest IDACI - Heterosexual/straight - Other (including mixed) | 0 | 11 | 3.32 | 1.07 | 7.17 | 3.33 | 1.17 | 6.85 | 0.00 | -1.62 | 1.75 |
| 22322 | Adolescent mental distress at either ages 15 or 17 - Female - Highest IDACI - Sexual minority - Black | 0 | 2 | 0.00 | 0.00 | 0.00 | 0.00 | 0.00 | 0.00 | 0.00 | 0.00 | 0.00 |
| 21322 | Adolescent mental distress at either ages 15 or 17 - Male - Highest IDACI - Sexual minority - Black | 0 | 1 | 0.00 | 0.00 | 0.00 | 0.00 | 0.00 | 0.00 | 0.00 | 0.00 | 0.00 |
| 21112 | Adolescent mental distress at either ages 15 or 17 - Male - Lowest IDACI - Heterosexual/straight - Black | 0 | 4 | 0.00 | 0.00 | 0.00 | 0.00 | 0.00 | 0.00 | 0.00 | 0.00 | 0.00 |
| 22222 | Adolescent mental distress at either ages 15 or 17 - Female - Middle IDACI - Sexual minority - Black | 0 | 2 | 0.00 | 0.00 | 0.00 | 0.00 | 0.00 | 0.00 | 0.00 | 0.00 | 0.00 |
| 21312 | Adolescent mental distress at either ages 15 or 17 - Male - Highest IDACI - Heterosexual/straight - Black | 0 | 11 | 0.00 | 0.00 | 0.00 | 0.00 | 0.00 | 0.00 | 0.00 | 0.00 | 0.00 |
| 12312 | No adolescent mental distress at either ages 15 or 17 - Female - Highest IDACI - Heterosexual/straight - Black | 0 | 26 | 0.00 | 0.00 | 0.00 | 0.00 | 0.00 | 0.00 | 0.00 | 0.00 | 0.00 |
| 11312 | No adolescent mental distress at either ages 15 or 17 - Male - Highest IDACI - Heterosexual/straight - Black | 0 | 23 | 0.00 | 0.00 | 0.00 | 0.00 | 0.00 | 0.00 | 0.00 | 0.00 | 0.00 |
| 12212 | No adolescent mental distress at either ages 15 or 17 - Female - Middle IDACI - Heterosexual/straight - Black | 0 | 12 | 0.00 | 0.00 | 0.00 | 0.00 | 0.00 | 0.00 | 0.00 | 0.00 | 0.00 |
| 11112 | No adolescent mental distress at either ages 15 or 17 - Male - Lowest IDACI - Heterosexual/straight - Black | 0 | 3 | 0.00 | 0.00 | 0.00 | 0.00 | 0.00 | 0.00 | 0.00 | 0.00 | 0.00 |
| 11212 | No adolescent mental distress at either ages 15 or 17 - Male - Middle IDACI - Heterosexual/straight - Black | 0 | 7 | 0.00 | 0.00 | 0.00 | 0.00 | 0.00 | 0.00 | 0.00 | 0.00 | 0.00 |
| 21212 | Adolescent mental distress at either ages 15 or 17 - Male - Middle IDACI - Heterosexual/straight - Black | 0 | 10 | 0.00 | 0.00 | 0.00 | 0.00 | 0.00 | 0.00 | 0.00 | 0.00 | 0.00 |
| 22212 | Adolescent mental distress at either ages 15 or 17 - Female - Middle IDACI - Heterosexual/straight - Black | 0 | 22 | 0.00 | 0.00 | 0.00 | 0.00 | 0.00 | 0.00 | 0.00 | 0.00 | 0.00 |
| 22112 | Adolescent mental distress at either ages 15 or 17 - Female - Lowest IDACI - Heterosexual/straight - Black | 0 | 11 | 0.00 | 0.00 | 0.00 | 0.00 | 0.00 | 0.00 | 0.00 | 0.00 | 0.00 |
| 22312 | Adolescent mental distress at either ages 15 or 17 - Female - Highest IDACI - Heterosexual/straight - Black | 0 | 45 | 0.00 | 0.00 | 0.00 | 0.00 | 0.00 | 0.00 | 0.00 | 0.00 | 0.00 |
| 12222 | No adolescent mental distress at either ages 15 or 17 - Female - Middle IDACI - Sexual minority - Black | 0 | 1 | 0.00 | 0.00 | 0.00 | 0.00 | 0.00 | 0.00 | 0.00 | 0.00 | 0.00 |
| 21222 | Adolescent mental distress at either ages 15 or 17 - Male - Middle IDACI - Sexual minority - Black | 0 | 1 | 0.00 | 0.00 | 0.00 | 0.00 | 0.00 | 0.00 | 0.00 | 0.00 | 0.00 |
| 11113 | No adolescent mental distress at either ages 15 or 17 - Male - Lowest IDACI - Heterosexual/straight - Asian | 0 | 33 | 1.00 | 0.31 | 2.16 | 1.00 | 0.34 | 2.07 | 0.00 | -0.48 | 0.50 |
| 11213 | No adolescent mental distress at either ages 15 or 17 - Male - Middle IDACI - Heterosexual/straight - Asian | 0 | 59 | 0.82 | 0.22 | 1.85 | 0.82 | 0.27 | 1.75 | 0.00 | -0.36 | 0.42 |
| 22313 | Adolescent mental distress at either ages 15 or 17 - Female - Highest IDACI - Heterosexual/straight - Asian | 3 | 88 | 3.40 | 1.43 | 6.28 | 3.40 | 1.39 | 6.25 | 0.00 | -1.44 | 1.57 |
| 12113 | No adolescent mental distress at either ages 15 or 17 - Female - Lowest IDACI - Heterosexual/straight - Asian | 0 | 17 | 1.29 | 0.36 | 2.90 | 1.28 | 0.45 | 2.66 | 0.01 | -0.59 | 0.65 |
| 11224 | No adolescent mental distress at either ages 15 or 17 - Male - Middle IDACI - Sexual minority - Other (including mixed) | 0 | 1 | 7.59 | 1.82 | 17.25 | 7.58 | 2.31 | 16.77 | 0.01 | -3.79 | 4.03 |
| 11114 | No adolescent mental distress at either ages 15 or 17 - Male - Lowest IDACI - Heterosexual/straight - Other (including mixed) | 0 | 9 | 2.63 | 0.71 | 6.02 | 2.62 | 0.92 | 5.49 | 0.02 | -1.24 | 1.55 |
| 21113 | Adolescent mental distress at either ages 15 or 17 - Male - Lowest IDACI - Heterosexual/straight - Asian | 0 | 12 | 2.67 | 0.84 | 5.93 | 2.66 | 0.98 | 5.42 | 0.02 | -1.31 | 1.45 |
| 22111 | Adolescent mental distress at either ages 15 or 17 - Female - Lowest IDACI - Heterosexual/straight - White | 19 | 385 | 4.87 | 3.22 | 6.81 | 4.85 | 3.04 | 7.07 | 0.02 | -1.75 | 1.90 |
| 11321 | No adolescent mental distress at either ages 15 or 17 - Male - Highest IDACI - Sexual minority - White | 0 | 3 | 5.54 | 1.78 | 12.63 | 5.52 | 2.11 | 11.72 | 0.03 | -2.73 | 3.59 |
| 12214 | No adolescent mental distress at either ages 15 or 17 - Female - Middle IDACI - Heterosexual/straight - Other (including mixed) | 0 | 9 | 2.76 | 0.81 | 6.35 | 2.72 | 0.97 | 5.56 | 0.04 | -1.24 | 1.53 |
| 12224 | No adolescent mental distress at either ages 15 or 17 - Female - Middle IDACI - Sexual minority - Other (including mixed) | 0 | 1 | 9.52 | 2.31 | 21.26 | 9.48 | 3.02 | 20.07 | 0.04 | -4.09 | 5.31 |
| 12314 | No adolescent mental distress at either ages 15 or 17 - Female - Highest IDACI - Heterosexual/straight - Other (including mixed) | 0 | 8 | 3.47 | 0.95 | 7.68 | 3.43 | 1.11 | 7.04 | 0.04 | -1.27 | 1.56 |
| 11323 | No adolescent mental distress at either ages 15 or 17 - Male - Highest IDACI - Sexual minority - Asian | 0 | 2 | 3.75 | 0.98 | 8.84 | 3.71 | 1.21 | 8.26 | 0.04 | -1.77 | 2.56 |
| 12313 | No adolescent mental distress at either ages 15 or 17 - Female - Highest IDACI - Heterosexual/straight - Asian | 1 | 50 | 1.34 | 0.44 | 3.00 | 1.30 | 0.45 | 2.61 | 0.05 | -0.44 | 0.76 |
| 11221 | No adolescent mental distress at either ages 15 or 17 - Male - Middle IDACI - Sexual minority - White | 0 | 6 | 4.43 | 1.40 | 9.49 | 4.38 | 1.70 | 8.80 | 0.05 | -2.07 | 2.30 |
| 11314 | No adolescent mental distress at either ages 15 or 17 - Male - Highest IDACI - Heterosexual/straight - Other (including mixed) | 0 | 7 | 2.76 | 0.71 | 6.50 | 2.71 | 0.85 | 5.94 | 0.05 | -1.17 | 1.82 |
| 12121 | No adolescent mental distress at either ages 15 or 17 - Female - Lowest IDACI - Sexual minority - White | 0 | 5 | 6.73 | 2.26 | 14.33 | 6.68 | 2.80 | 12.79 | 0.05 | -3.23 | 3.91 |
| 21221 | Adolescent mental distress at either ages 15 or 17 - Male - Middle IDACI - Sexual minority - White | 1 | 10 | 10.95 | 4.67 | 20.18 | 10.89 | 5.29 | 18.74 | 0.06 | -4.78 | 5.58 |
| 12213 | No adolescent mental distress at either ages 15 or 17 - Female - Middle IDACI - Heterosexual/straight - Asian | 1 | 34 | 1.12 | 0.36 | 2.61 | 1.04 | 0.34 | 2.19 | 0.07 | -0.32 | 0.93 |
| 11223 | No adolescent mental distress at either ages 15 or 17 - Male - Middle IDACI - Sexual minority - Asian | 0 | 4 | 3.11 | 0.72 | 7.78 | 3.03 | 0.91 | 6.80 | 0.08 | -1.33 | 2.05 |
| 22321 | Adolescent mental distress at either ages 15 or 17 - Female - Highest IDACI - Sexual minority - White | 1 | 6 | 16.53 | 7.00 | 29.87 | 16.42 | 7.74 | 28.17 | 0.11 | -6.21 | 8.43 |
| 21224 | Adolescent mental distress at either ages 15 or 17 - Male - Middle IDACI - Sexual minority - Other (including mixed) | 0 | 1 | 17.79 | 6.23 | 37.34 | 17.67 | 7.13 | 33.93 | 0.12 | -6.94 | 9.80 |
| 11214 | No adolescent mental distress at either ages 15 or 17 - Male - Middle IDACI - Heterosexual/straight - Other (including mixed) | 1 | 11 | 2.29 | 0.70 | 5.35 | 2.15 | 0.72 | 4.41 | 0.14 | -0.72 | 1.84 |
| 11111 | No adolescent mental distress at either ages 15 or 17 - Male - Lowest IDACI - Heterosexual/straight - White | 6 | 242 | 1.61 | 0.82 | 2.91 | 1.46 | 0.71 | 2.53 | 0.15 | -0.36 | 1.28 |
| 11211 | No adolescent mental distress at either ages 15 or 17 - Male - Middle IDACI - Heterosexual/straight - White | 4 | 138 | 1.38 | 0.61 | 2.88 | 1.21 | 0.52 | 2.16 | 0.17 | -0.25 | 1.45 |
| 21123 | Adolescent mental distress at either ages 15 or 17 - Male - Lowest IDACI - Sexual minority - Asian | 0 | 1 | 9.37 | 2.93 | 21.13 | 9.20 | 3.37 | 19.22 | 0.18 | -3.73 | 6.02 |
| 22114 | Adolescent mental distress at either ages 15 or 17 - Female - Lowest IDACI - Heterosexual/straight - Other (including mixed) | 2 | 21 | 8.55 | 3.52 | 16.04 | 8.34 | 3.57 | 14.85 | 0.21 | -2.93 | 4.64 |
| 22324 | Adolescent mental distress at either ages 15 or 17 - Female - Highest IDACI - Sexual minority - Other (including mixed) | 1 | 3 | 25.51 | 9.59 | 46.66 | 25.30 | 10.51 | 44.22 | 0.21 | -8.55 | 10.30 |
| 12324 | No adolescent mental distress at either ages 15 or 17 - Female - Highest IDACI - Sexual minority - Other (including mixed) | 0 | 1 | 11.79 | 3.41 | 26.69 | 11.57 | 3.56 | 23.32 | 0.22 | -4.73 | 7.70 |
| 11121 | No adolescent mental distress at either ages 15 or 17 - Male - Lowest IDACI - Sexual minority - White | 1 | 11 | 5.54 | 2.03 | 11.98 | 5.27 | 2.28 | 9.93 | 0.27 | -1.82 | 5.41 |
| 22211 | Adolescent mental distress at either ages 15 or 17 - Female - Middle IDACI - Heterosexual/straight - White | 10 | 194 | 4.30 | 2.42 | 6.92 | 4.03 | 2.19 | 6.42 | 0.27 | -1.29 | 2.68 |
| 22311 | Adolescent mental distress at either ages 15 or 17 - Female - Highest IDACI - Heterosexual/straight - White | 4 | 57 | 5.40 | 2.37 | 10.22 | 5.12 | 2.28 | 9.17 | 0.28 | -1.70 | 3.83 |
| 21214 | Adolescent mental distress at either ages 15 or 17 - Male - Middle IDACI - Heterosexual/straight - Other (including mixed) | 1 | 8 | 5.90 | 2.11 | 12.63 | 5.55 | 2.24 | 11.32 | 0.35 | -1.68 | 4.74 |
| 12323 | No adolescent mental distress at either ages 15 or 17 - Female - Highest IDACI - Sexual minority - Asian | 1 | 2 | 5.07 | 1.55 | 11.93 | 4.69 | 1.55 | 10.61 | 0.38 | -1.58 | 4.46 |
| 22214 | Adolescent mental distress at either ages 15 or 17 - Female - Middle IDACI - Heterosexual/straight - Other (including mixed) | 2 | 17 | 7.29 | 2.95 | 14.54 | 6.91 | 3.00 | 13.22 | 0.38 | -2.31 | 4.57 |
| 12321 | No adolescent mental distress at either ages 15 or 17 - Female - Highest IDACI - Sexual minority - White | 1 | 4 | 7.40 | 2.53 | 16.62 | 6.95 | 2.71 | 14.14 | 0.45 | -2.21 | 5.28 |
| 21314 | Adolescent mental distress at either ages 15 or 17 - Male - Highest IDACI - Heterosexual/straight - Other (including mixed) | 2 | 12 | 7.40 | 2.56 | 16.55 | 6.92 | 2.62 | 13.83 | 0.48 | -2.36 | 6.13 |
| 21223 | Adolescent mental distress at either ages 15 or 17 - Male - Middle IDACI - Sexual minority - Asian | 1 | 2 | 8.33 | 2.68 | 18.70 | 7.66 | 2.64 | 16.00 | 0.67 | -2.51 | 6.73 |
| 22113 | Adolescent mental distress at either ages 15 or 17 - Female - Lowest IDACI - Heterosexual/straight - Asian | 5 | 37 | 4.11 | 1.66 | 9.35 | 3.35 | 1.31 | 6.36 | 0.76 | -0.41 | 5.77 |
| 21321 | Adolescent mental distress at either ages 15 or 17 - Male - Highest IDACI - Sexual minority - White | 2 | 5 | 14.33 | 5.91 | 29.10 | 13.41 | 5.78 | 24.73 | 0.92 | -4.01 | 12.44 |
| 22224 | Adolescent mental distress at either ages 15 or 17 - Female - Middle IDACI - Sexual minority - Other (including mixed) | 1 | 2 | 22.36 | 8.53 | 42.40 | 21.36 | 9.36 | 38.19 | 1.01 | -6.05 | 12.99 |
| 21324 | Adolescent mental distress at either ages 15 or 17 - Male - Highest IDACI - Sexual minority - Other (including mixed) | 1 | 1 | 22.43 | 8.05 | 47.09 | 21.13 | 8.19 | 38.92 | 1.30 | -5.88 | 17.46 |

*Note.* I+M effects = interaction effects and main effects conflated; M effects = main effects only; I effects = differences between intersections and main effects.
